# Supplementary material for: DeepRTAlign: toward accurate retention time alignment for large cohort mass spectrometry data analysis
Source: Nat Commun. 2023 Dec 11;14:8188. doi: 10.1038/s41467-023-43909-5 (PMC10713976; doi:10.1038/s41467-023-43909-5)
Supplement: Supplementary file 1 — Supplementary Information [file 41467_2023_43909_MOESM1_ESM.pdf]

Supplementary Information of

DeepRTAlign: toward accurate retention time alignment for large cohort mass spectrometry data analysis

Yi Liu[1,2,#], Yun Yang[3,4,#], Wendong Chen[3,4], Feng Shen[5], Linhai Xie[2,3,4], Yingying Zhang[2,6], Yuanjun Zhai[2], Fuchu He[2,7], Yunping Zhu[2,*], Cheng Chang[2,7,*]

1. Faculty of Environment and Life, Beijing University of Technology, Beijing 100023, China.
2. State Key Laboratory of Proteomics, Beijing Proteome Research Center, National Center for Protein Sciences (Beijing), Beijing Institute of Lifeomics, Beijing 102206, China.
3. International Academy of Phronesis Medicine (Guang Dong), No. 96 Xindao Ring South Road, Guangzhou International Bio Island, Guangzhou 510000, China
4. South China Institute of Biomedicine, No. 83 Ruihe Road, Guangzhou 510535, China
5. Department of Hepatic Surgery IV, the Eastern Hepatobiliary Surgery Hospital, Naval Medical University, Shanghai 200433, China
6. Chongqing Key Laboratory on Big Data for Bio Intelligence, Chongqing University of Posts and Telecommunications, Chongqing 400065, China
7. Research Unit of Proteomics Driven Cancer Precision Medicine, Chinese Academy of Medical Sciences, Beijing 102206, China.

[#]These authors contributed equally: Yi Liu and Yun Yang

* To whom correspondence should be addressed:

Yunping Zhu, Email: zhuyunping@gmail.com

Cheng Chang, Email: changchengbio@163.com

TOC

Supplementary Tables 3
Supplementary Figures 8

Supplementary Tables

Supplementary Table 1. The datasets used for training and testing the deep learning model of DeepRTAlign in this study. The sample numbers in this table were the number of samples used in this work. HCC-T and HCC-N indicated the data from tumor and non-tumor samples of an HCC cohort (N=101). HCC-R and HCC-R2 were data from two HCC cohorts. UPS2-M and UPS2-Y were two benchmark datasets from mouse cells and yeast cells with UPS2 proteins spiked in. EC-H was a dataset from the mixture of human cells and E. coli cells. AT was a dataset based on the *Arabidopsis thaliana* seeds. SC was a single-cell proteomic dataset. MI was based on mouse intestinal samples. CD was obtained from the gut microbiota of patients with Crohn's disease. NCC19, SM1100, MM, SO and GUS were public metabolomic datasets. Benchmark-QC-H and Benchmark-QC-E were two benchmark datasets based on HEK 293T and E. coli samples, respectively. Benchmark-FC was a benchmark dataset with known fold changes. Benchmark-RT contained two HEK 293T samples with different RT gradients (60 min and 120 min). Benchmark-MV was a benchmark dataset containing different proportions of HEK 293T and E. coli samples from six Orbitrap Exploris 480 instruments.

Dataset name	Sample numbers	Dataset ID	RT range (min)	Type
HCC-T	101	PXD006512	80	Training set
HCC-N	101	PXD006512	80	Proteomic test set
HCC-R	11	PXD022881	60	Proteomic test set
UPS2-M	12	PXD008428	100	Proteomic test set
UPS2-Y	12	PXD008428	100	Proteomic test set
EC-H	20	PXD003881	170	Proteomic test set
AT	18	PXD027546	130	Proteomic test set
SC	18	PXD025634	90	Proteomic test set
MI	1	PXD002838	180	Proteomic test set
CD	1	PXD002882	120	Proteomic test set
NCC19	1	MTBLS1866	30	Metabolomic test set
SM1100	10	MTBLS733	50	Metabolomic test set
MM	1	MTBLS5430	40	Metabolomic test set
SO	1	MTBLS492	45	Metabolomic test set
GUS	1	MTBLS650	40	Metabolomic test set
HCC-R2	23	IPX0006622000	180	PRM validation
Benchmark-FC	12	IPX0006638000	60	Benchmark (known fold changes)
Benchmark-QC-H	3	IPX0006819000	60	Benchmark for QC
Benchmark-QC-E	3	IPX0006819000	60	Benchmark for QC
Benchmark-RT	2	IPX0006820000	60 and 120	Alignment for different gradients
Benchmark-MV	24	IPX0007319000	60	Benchmark for reducing missing values

Supplementary Table 2. Parameters optimization for the DNN model in DeepRTAlign based on the 10-fold cross validation results of the training set HCC-T.

(a) Optimization for hidden layer number in the DNN model. In this test, each layer has 5000 neurons.

Hidden layer number	1	2	3	4	5
AUC	0.988±0.003	0.990±0.002	**0.993±0.002**	0.992±0.003	0.993±0.002

(b) Optimization for neuron number in the DNN model. All the models have 3 hidden layers.

Neuron number	50	500	5000	50000	500000
AUC	0.887±0.012	0.969±0.011	**0.993±0.002**	0.993±0.001	0.992±0.001

Supplementary Table 3. The AUCs on different test sets. All the results are based on the model trained on the HCC-T dataset. In each test set, we randomly selected 10,000 positive and 10,000 negative feature pairs to perform this evaluation.

Dataset	DNN	RF	KNN	SVM	LR
HCC-N	**0.925**	0.916	0.656	0.865	0.894
HCC-R	**0.933**	0.905	0.668	0.901	0.899
UPS2-M	**0.979**	0.919	0.683	0.896	0.905
UPS2-Y	**0.971**	0.920	0.702	0.900	0.897
EC-H	**0.972**	0.938	0.733	0.912	0.944
AT	**0.975**	0.943	0.785	0.932	0.945
SC	**0.917**	0.901	0.752	0.842	0.898

Supplementary Table 4. The AUCs of DeepRTAlign when using different samples in the test sets as the anchor sample. All the results are based on the model trained on the HCC-T dataset. In each test set, five samples are randomly selected.

Dataset	Sample 1	Sample 2	Sample 3	Sample 4	Sample 5
HCC-N	0.925	0.926	0.925	0.926	0.924
HCC-R	0.933	0.930	0.930	0.933	0.934
UPS2-M	0.979	0.977	0.976	0.976	0.981
UPS2-Y	0.971	0.972	0.973	0.971	0.971
EC-H	0.972	0.971	0.972	0.972	0.972
AT	0.975	0.975	0.974	0.976	0.975
SC	0.917	0.909	0.915	0.919	0.918

Supplementary Table 5. The AUCs of DeepRTAlign with or without coarse alignment step in different test sets. All the results are based on the model trained on HCC-T dataset. And in this table, all the models have 3 hidden layers, and each layer has 5000 neurons.

Dataset	With coarse alignment	Without coarse alignment
HCC-N	**0.925**	0.899
HCC-R	**0.933**	0.875
UPS2-M	**0.979**	0.909
UPS2-Y	**0.971**	0.898
EC-H	**0.972**	0.905
AT	**0.975**	0.917
SC	**0.917**	0.821

Supplementary Table 6. The list of feature importance of the DNN model, the RF model and the LR model. The DNN model, the RF model and the LR model were trained on the same training set (HCC-T dataset). Please note that the feature importance of LR model is ranked by the absolute value of "coef_".

DNN			RF			LR		
index	importance	Features	index	importance	Features	index	importance	Features
16	0.107	mz_m-mz_n	16	0.205	mz_m-mz_n	16	8.804	mz_m-mz_n
6	0.107	mz_n-mz_m	6	0.199	mz_n-mz_m	6	8.803	mz_n-mz_m
5	0.048	RT_n-RT_m	5	0.065	RT_n-RT_m	27	-2.435	RT_{n+1}
15	0.048	RT_m-RT_n	15	0.058	RT_m-RT_n	21	-2.294	RT_{n-2}
11	0.026	RT_{m-2}-RT_n	11	0.030	RT_{m-2}-RT_n	25	1.129	RT_n
13	0.024	RT_{m-1}-RT_n	35	0.025	RT_m	37	0.928	RT_{m+1}
1	0.023	RT_{n-2}-RT_m	25	0.023	RT_n	29	0.854	RT_{n+2}
17	0.017	RT_{m+1}-RT_n	13	0.018	RT_{m-1}-RT_n	31	0.679	RT_{m-2}
10	0.010	mz_{n+2}-mz_m	19	0.017	RT_{m+2}-RT_n	23	0.415	RT_{n-1}
4	0.010	mz_{n-1}-mz_m	17	0.016	RT_{m+1}-RT_n	35	0.397	RT_m
31	0.006	RT_{m-2}	37	0.016	RT_{m+1}	17	0.234	RT_{m+1}-RT_n
21	0.006	RT_{n-2}	27	0.015	RT_{n+1}	32	-0.216	mz_{m-2}
8	0.005	mz_{n+1}-mz_m	39	0.015	RT_{m+2}	30	-0.216	mz_{n+2}
14	0.005	mz_{m-1}-mz_n	7	0.014	RT_{n+1}-RT_m	34	-0.216	mz_{m-1}
9	0.004	RT_{n+2}-RT_m	9	0.013	RT_{n+2}-RT_m	38	-0.216	mz_{m+1}
3	0.002	RT_{n-1}-RT_m	36	0.013	mz_m	24	-0.216	mz_{n-1}
7	0.002	RT_{n+1}-RT_m	23	0.013	RT_{n-1}	40	-0.216	mz_{m+2}
18	0.002	mz_{m+1}-mz_n	12	0.012	mz_{m-2}-mz_n	36	-0.216	mz_m
34	0.002	mz_{m-1}	1	0.012	RT_{n-2}-RT_m	26	-0.216	mz_n
20	0.001	mz_{m+2}-mz_n	14	0.012	mz_{m-1}-mz_n	22	-0.216	mz_{n-2}
36	0.001	mz_m	3	0.012	RT_{n-1}-RT_m	28	-0.216	mz_{n+1}
22	0.001	mz_{n-2}	10	0.012	mz_{n+2}-mz_m	18	-0.210	mz_{m+1}-mz_n
33	0.001	RT_{m-1}	32	0.012	mz_{m-2}	4	-0.185	mz_{n-1}-mz_m

38	0.001	mz_{m+1}	34	0.012	mz_{m-1}	11	-0.163	$RT_{m-2}-RT_{n}$
19	0.001	$RT_{m+2}-RT_{n}$	21	0.011	RT_{n-2}	19	-0.141	$RT_{m+2}-RT_{n}$
28	0.001	mz_{n+1}	38	0.011	mz_{m+1}	33	-0.101	RT_{m-1}
40	0.001	mz_{m+2}	18	0.011	$mz_{m+1}-mz_{n}$	20	0.080	$mz_{m+2}-mz_{n}$
26	0.001	mz_{n}	22	0.011	mz_{n-2}	7	-0.080	$RT_{n+1}-RT_{m}$
30	0.000	mz_{n+2}	29	0.011	RT_{n+2}	8	-0.074	$mz_{n+1}-mz_{m}$
32	0.000	mz_{m-2}	30	0.010	mz_{n+2}	14	-0.073	$mz_{m-1}-mz_{n}$
25	0.000	RT_{n}	2	0.010	$mz_{n-2}-mz_{m}$	5	0.063	$RT_{n}-RT_{m}$
23	0.000	RT_{n-1}	33	0.010	RT_{m-1}	15	0.063	$RT_{m}-RT_{n}$
24	0.000	mz_{n-1}	20	0.010	$mz_{m+2}-mz_{n}$	39	0.058	RT_{m+2}
2	0.000	$mz_{n-2}-mz_{m}$	8	0.010	$mz_{n+1}-mz_{m}$	9	-0.045	$RT_{n+2}-RT_{m}$
12	0.000	$mz_{m-2}-mz_{n}$	40	0.010	mz_{m+2}	2	-0.040	$mz_{n-2}-mz_{m}$
27	0.000	RT_{n+1}	24	0.010	mz_{n-1}	1	0.036	$RT_{n-2}-RT_{m}$
29	0.000	RT_{n+2}	26	0.009	mz_{n}	13	0.036	$RT_{m-1}-RT_{n}$
35	0.000	RT_{m}	28	0.009	mz_{n+1}	10	-0.026	$mz_{n+2}-mz_{m}$
37	0.000	RT_{m+1}	31	0.009	RT_{m-2}	12	-0.023	$mz_{m-2}-mz_{n}$
39	0.000	RT_{m+2}	4	0.009	$mz_{n-1}-mz_{m}$	3	0.010	$RT_{n-1}-RT_{m}$

Supplementary Table 7. The minimum information required for alignment in each tool. Symbol "√" represents for required and "-" represents for "not required".

Tools	MS	MS/MS	Identification results
DeepRTAlign	√	-	-
MZmine 2	√	-	-
OpenMS	√	-	-
Quandenser	√	√	-
MaxQuant	√	√	√
MSFragger	√	√	√
DIA-NN	√	√	√

Supplementary Table 8. The different algorithm combinations for benchmarking DeepRTAlign against MZmine 2 and OpenMS on a public metabolomic test set SM1100.

Abbreviations	Feature extraction	Feature alignment	Precision	Recall
MM	MZmine 2	MZmine 2	1.000	1.000
MD	MZmine 2	DeepRTAlign	1.000	1.000
OO	OpenMS	OpenMS	1.000	0.980
OD	OpenMS	DeepRTAlign	0.997	0.985
DD	Dinosaur	DeepRTAlign	0.971	0.965

Supplementary Table 9. Parameters optimization for K in the KNN model based on the 10-fold cross validation results of the training set HCC-T. All the other parameters were kept default in scikit-learn v0.21.3.

K	1	2	3	4	5	6
AUC	0.807±0.080	0.836±0.085	0.850±0.083	0.853±0.083	**0.853±0.081**	0.852±0.077

Supplementary Table 10. Parameters optimization in the LR model based on the 10-fold cross validation results of the training set HCC-T. All the other parameters were kept default in scikit-learn v0.21.3.

(a) Optimization for solver in the LR model. All the other parameters were kept default in scikit-learn v0.21.3.

solver	lbfgs	liblinear	newton-cg	sag	saga
AUC	**0.912±0.018**	0.911±0.017	0.911±0.017	0.911±0.017	0.911±0.017

(b) Optimization for penalty in the LR model. The solver was set to "lbfgs". All the other parameters were kept default in scikit-learn v0.21.3.

penalty	L2	None
AUC	**0.911±0.017**	0.911±0.017

Supplementary Figures

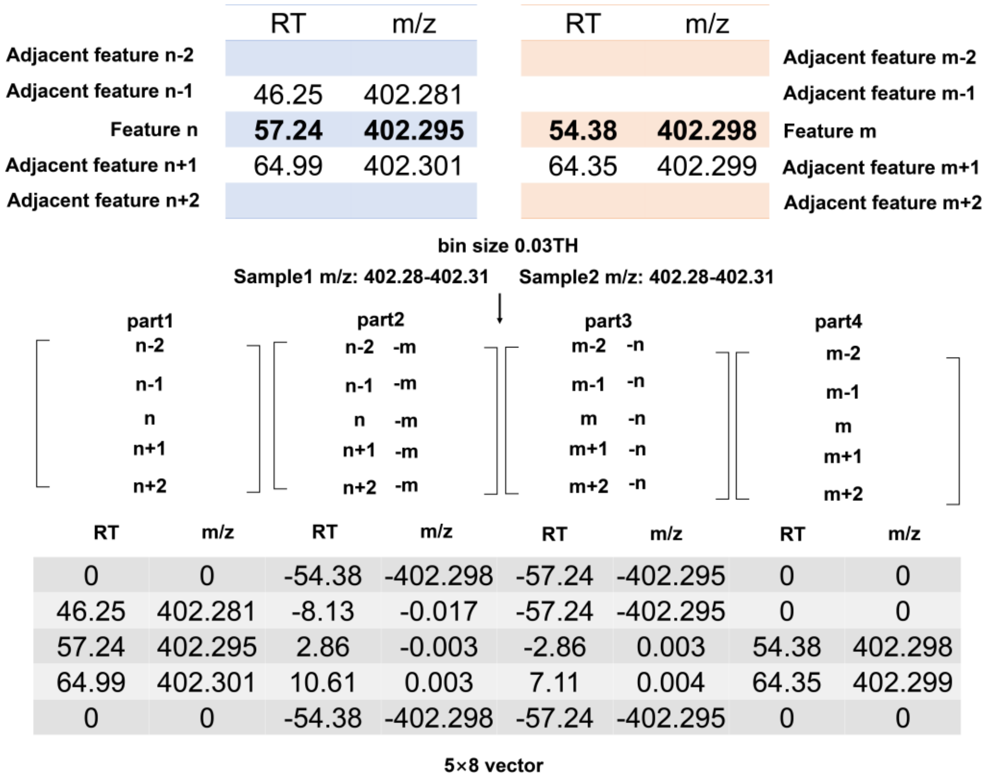

Supplementary Fig. 1. An input example for DeepRTAlign. After min-max normalization on each column, this 5×8 vector is used as the input to the neural network. If feature n and feature m are the same peptide, this vector will be labeled as "aligned" (should be aligned), otherwise it will be labeled as "non-aligned" (should not be aligned).

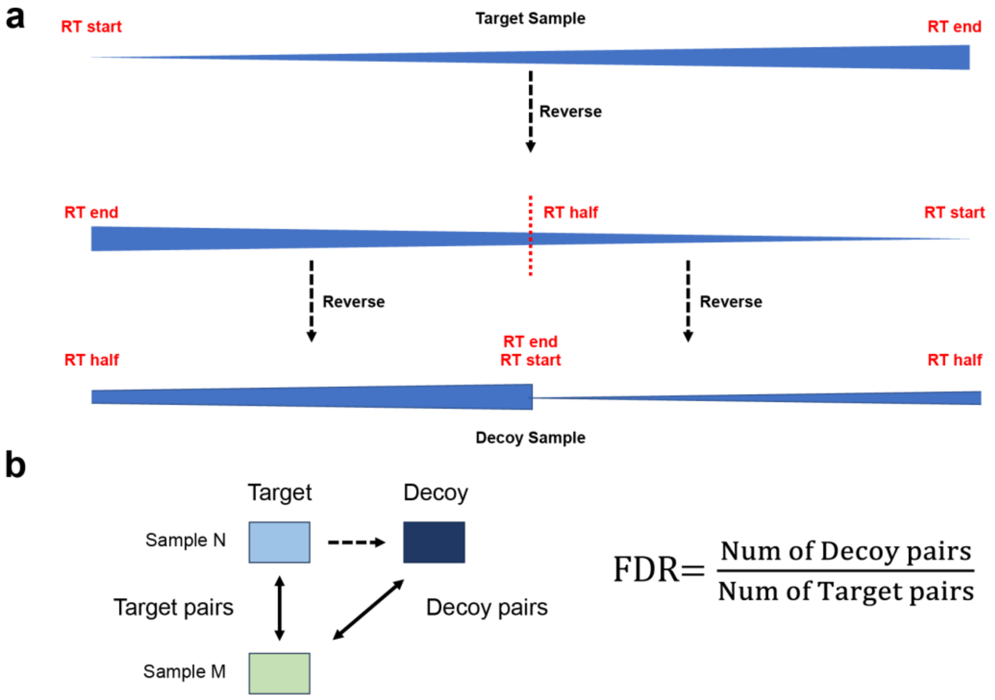

Supplementary Fig. 2. Illustration of the QC module in DeepRTAlign. a The decoy design workflow. **b** The FDR calculation workflow.

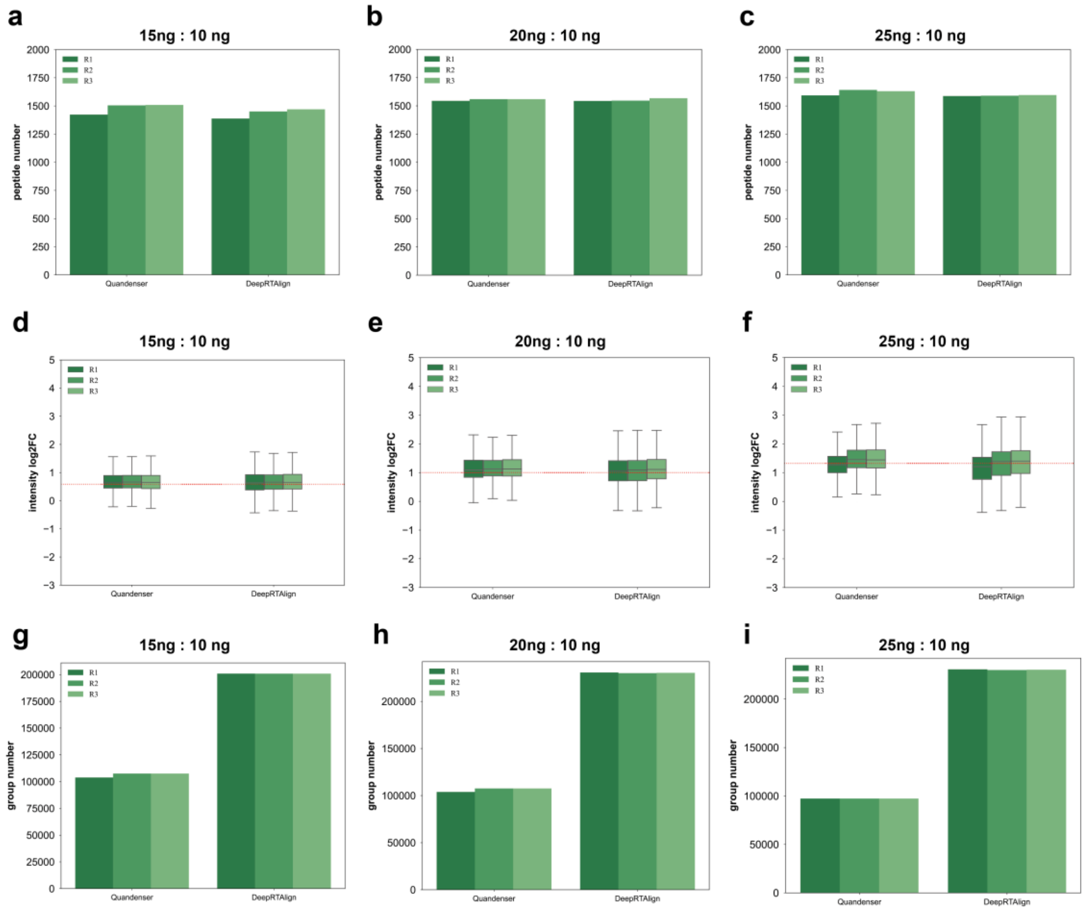

Supplementary Fig. 3. **Comparison of DeepRTAlign and Quandenser on Benchmark-FC dataset.** The number and ratio distributions of all E. coli peptides and the group number of aligned features between specific samples (**a**, **d**, **g**: 15ng/10ng, **b**, **e**, **h**: 20ng/10ng, and **c**, **f**, **i**: 25ng/10ng) in each replicate (R1, R2 and R3) after alignment by Quandenser and DeepRTAlign. It should be noted that a group is defined as a set of aligned features in different runs. Source data are provided as a Source Data file.

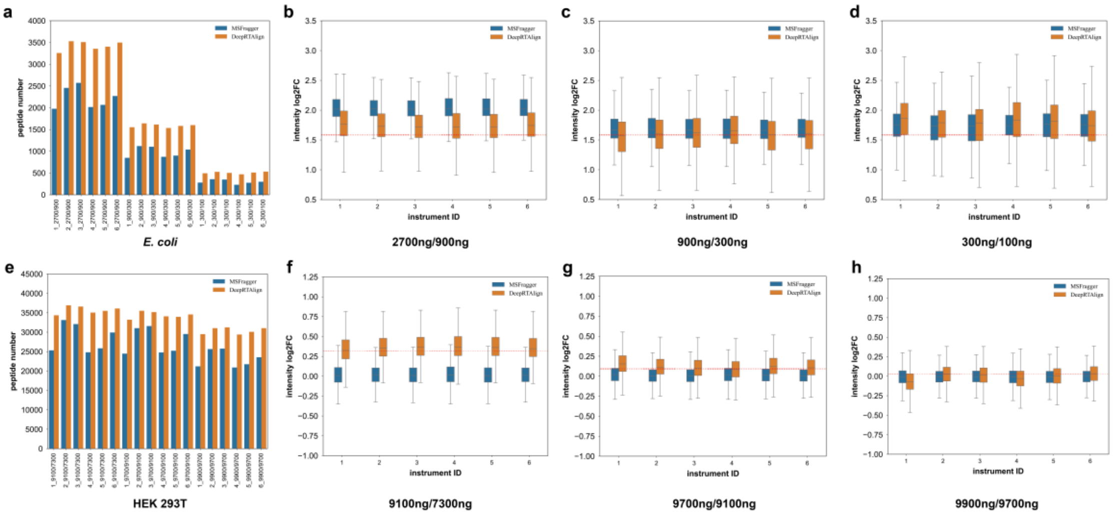

Supplementary Fig. 4. **Comparison of DeepRTAlign and MSFragger on Benchmark-MV dataset. a, e** Feature numbers corresponding to the *E. coli* peptides and the HEK 293T peptides

identified in dataset Benchmark-MV by MSFragger with match between runs (MBR) and DeepRTAlign, respectively. **b-d** and **f-h** Ratio boxplots for the features corresponding to the *E. coli* peptides or the HEK 293T peptides identified in dataset Benchmark-MV by MSFragger with MBR and DeepRTAlign, respectively. The orange dashed line indicates the theoretical ratio. For DeepRTAlign results, features were extracted by Dinosaur, and then aligned by DeepRTAlign. MSFragger's identification results were used to match these features (mass tolerance: ± 10 ppm, RT tolerance: restrict the RT of a peptide to be within the RT range of the corresponding precursor feature). Source data are provided as a Source Data file.

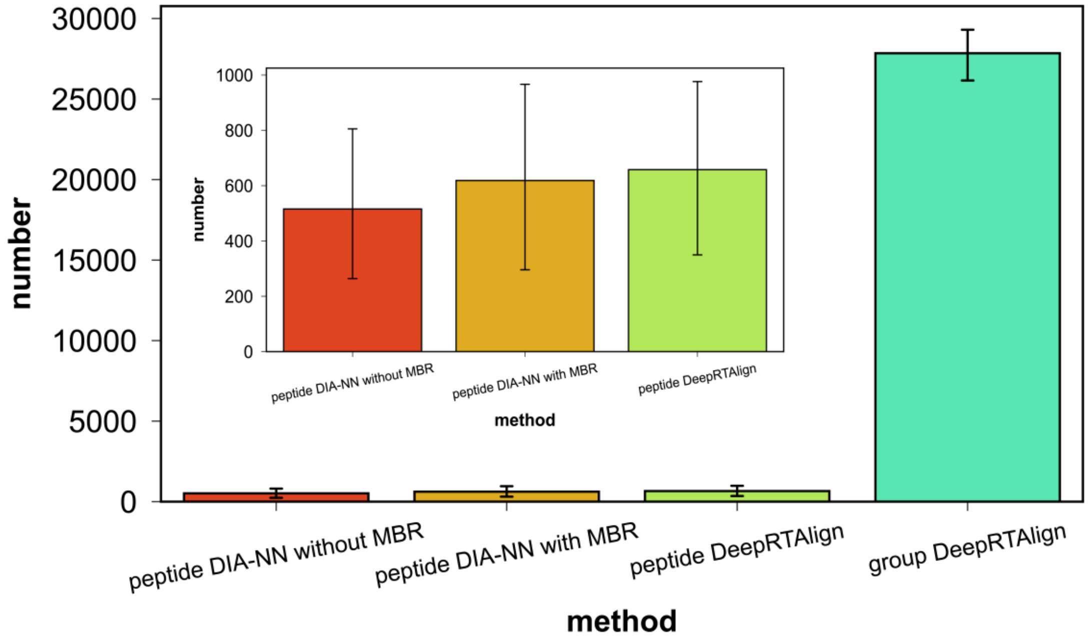

Supplementary Fig. 5. The peptide number and feature number of each HT22 cell. Features are extracted by Dinosaur. Only the features presented in at least two cells are considered. MBR: match between runs. Error bar indicates standard deviation. It should be noted that a group is defined as a set of aligned features in different runs. Source data are provided as a Source Data file.

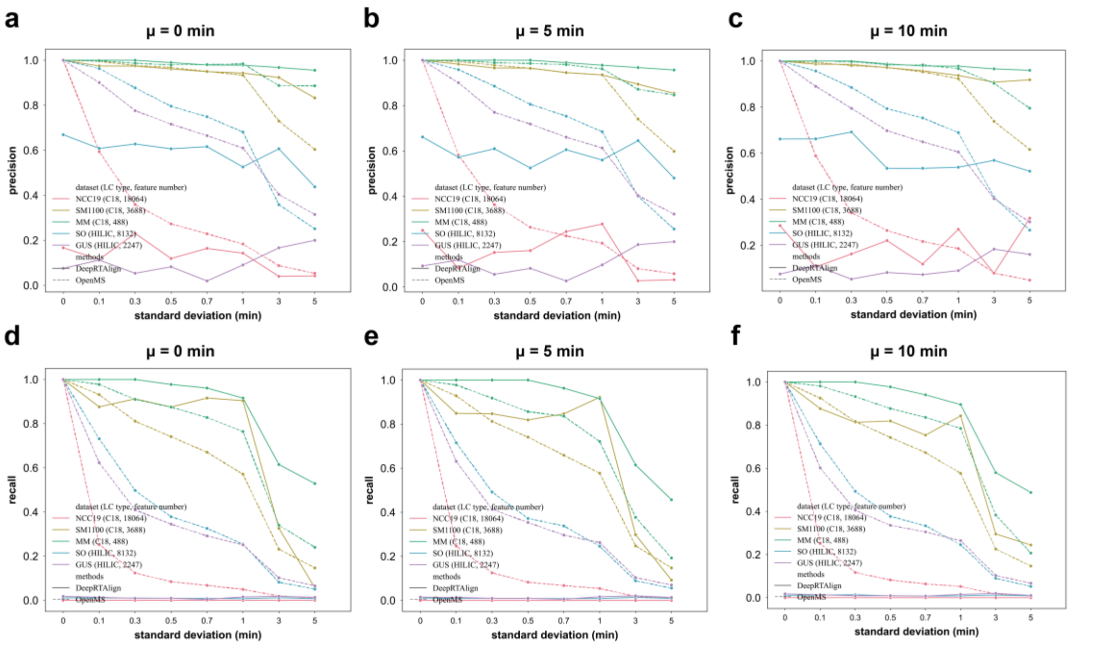

Supplementary Fig. 6. **Comparison of DeepRTAlign and OpenMS on multiple simulated datasets generated from 5 real-world metabolomic datasets.** The simulated datasets were constructed by adding normally distributed RT shifts to the corresponding real-world dataset. (**a**, **d**) μ=0 min. (**b**, **e**) μ=5 min. (**c**, **f**) μ=10 min. The normal distribution has an increasing σ, i.e., σ=0, 0.1, 0.3, 0.5, 0.7, 1, 3, 5 for different μ (0, 5 and 10 minutes), respectively. The FDR of DeepRTAlign's results is set to 1%. Source data are provided as a Source Data file.

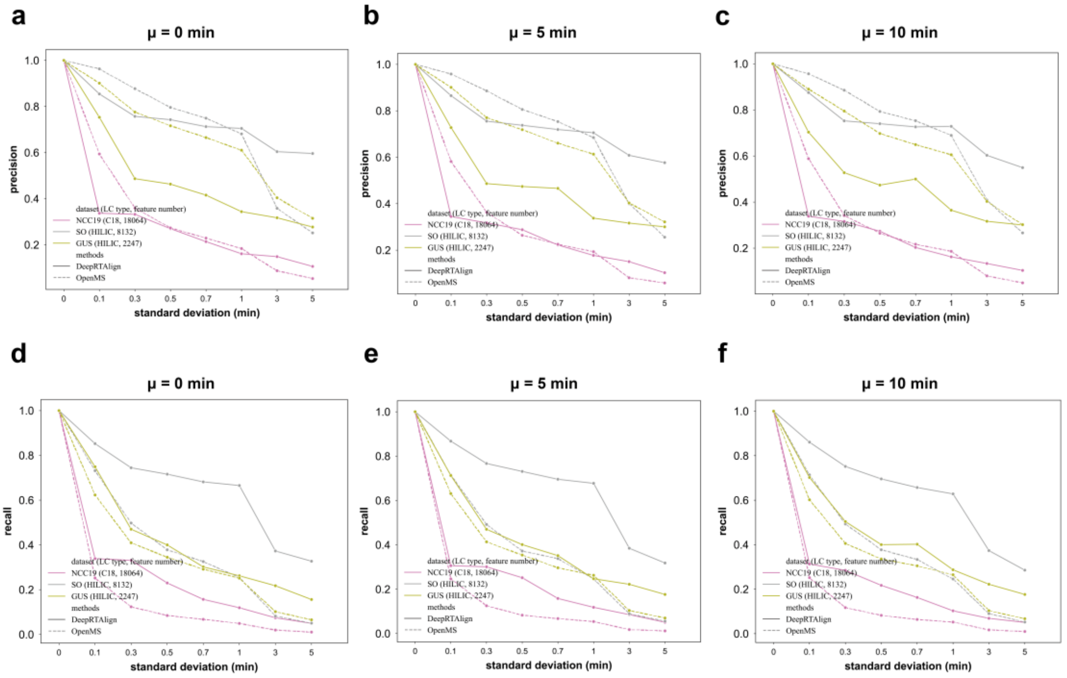

Supplementary Fig. 7. **Comparison of DeepRTAlign and OpenMS on multiple simulated datasets generated from 3 real-world metabolomic datasets.** The simulated datasets were constructed by adding normally distributed RT shifts to the corresponding real-world dataset. (**a**, **d**) μ=0 min. (**b**, **e**) μ=5 min. (**c**, **f**) μ=10 min. The normal distribution has an increasing σ, i.e., σ=0, 0.1,

0.3, 0.5, 0.7, 1, 3, 5 for different μ (0, 5 and 10 minutes), respectively. The FDR of DeepRTAlign's results is set to 100%. Source data are provided as a Source Data file.

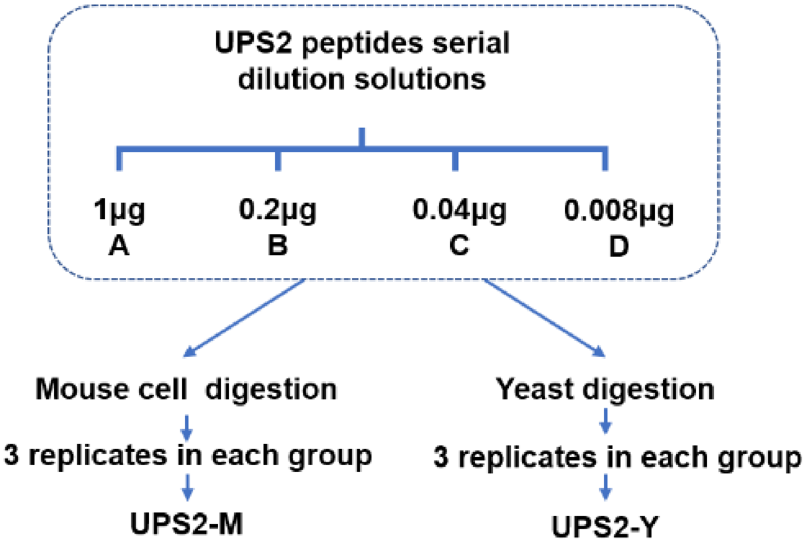

Supplementary Fig. 8. Experimental design of UPS2-Y and UPS2-M data sets. A series of UPS2 protein digestions (1, 0.2, 0.04, and 0.008 μg, represented as A, B, C, and D in this study) was added into an equal amount of mouse cell and yeast mixtures to build the UPS2-M and UPS2-Y datasets. This figure was modified from our previous paper (Chang et al. Anal Chem 2016, 88 (13), 6844–6851).
