# Supplementary material for: DeepRTAlign: toward accurate retention time alignment for large cohort mass spectrometry data analysis
Source: Nat Commun. 2023 Dec 11;14:8188. doi: 10.1038/s41467-023-43909-5 (PMC10713976; doi:10.1038/s41467-023-43909-5)
Supplement: Supplementary file 2 — Reporting Summary [file 41467_2023_43909_MOESM2_ESM.pdf]

nature portfolio

Corresponding author(s): Cheng Chang & Yunping Zhu

Last updated by author(s): Nov 18, 2023

# Reporting Summary

Nature Portfolio wishes to improve the reproducibility of the work that we publish. This form provides structure for consistency and transparency in reporting. For further information on Nature Portfolio policies, see our Editorial Policies and the Editorial Policy Checklist.

## Statistics

For all statistical analyses, confirm that the following items are present in the figure legend, table legend, main text, or Methods section.

| n/a | Confirmed | |
|---|---|---|
| x | | The exact sample size ($n$) for each experimental group/condition, given as a discrete number and unit of measurement |
| | x | A statement on whether measurements were taken from distinct samples or whether the same sample was measured repeatedly |
| | x | The statistical test(s) used AND whether they are one- or two-sided<br>*Only common tests should be described solely by name; describe more complex techniques in the Methods section.* |
| x | | A description of all covariates tested |
| x | | A description of any assumptions or corrections, such as tests of normality and adjustment for multiple comparisons |
| | x | A full description of the statistical parameters including central tendency (e.g. means) or other basic estimates (e.g. regression coefficient) AND variation (e.g. standard deviation) or associated estimates of uncertainty (e.g. confidence intervals) |
| | x | For null hypothesis testing, the test statistic (e.g. $F$, $t$, $r$) with confidence intervals, effect sizes, degrees of freedom and $P$ value noted<br>*Give P values as exact values whenever suitable.* |
| x | | For Bayesian analysis, information on the choice of priors and Markov chain Monte Carlo settings |
| x | | For hierarchical and complex designs, identification of the appropriate level for tests and full reporting of outcomes |
| x | | Estimates of effect sizes (e.g. Cohen's $d$, Pearson's $r$), indicating how they were calculated |

*Our web collection on statistics for biologists contains articles on many of the points above.*

## Software and code

Policy information about availability of computer code

| | |
|---|---|
| Data collection | No software was used for data collection. |
| Data analysis | All the codes of DeepRTAlign are programmed in Python v3.7.4. Numpy v1.16.4 is used to preprocess data. SciPy v1.6.2 is used for statistical methods. Pytorch framework v1.8.0 is used to implement the DNN model, and scikit-learn framework v0.21.3 is used to implement other machine learning algorithms. Mrmr-selection v0.2.3 is used for mRMR algorithm. All the source codes of DeepRTAlign (including the feature extraction tool XICFinder) are freely available from GitHub (https://github.com/PHOENIXcenter/deeprtalign) under GNU General Public License version 3.0. |

For manuscripts utilizing custom algorithms or software that are central to the research but not yet described in published literature, software must be made available to editors and reviewers. We strongly encourage code deposition in a community repository (e.g. GitHub). See the Nature Portfolio guidelines for submitting code & software for further information.

## Data

Policy information about availability of data

All manuscripts must include a data availability statement. This statement should provide the following information, where applicable:

- Accession codes, unique identifiers, or web links for publicly available datasets
- A description of any restrictions on data availability
- For clinical datasets or third party data, please ensure that the statement adheres to our policy

Datasets HCC-T and HCC-N can be downloaded from the iProX database under accession number IPX0000937000[https://www.iprox.cn//page/project.html?

id=IPX0000937000] or PXD006512[https://www.ebi.ac.uk/pride/archive/projects/PXD006512].
Datasets UPS2-M and UPS2-Y can be downloaded from the iProX database under the accession number IPX00075500[https://www.iprox.cn//page/project.html?id=IPX0000755000] or PXD008428[https://proteomecentral.proteomexchange.org/cgi/GetDataset?ID=PXD008428].
Datasets HCC-R, EC-H, AT, SC, MI and CD can be downloaded from the PRIDE database under the accession numbers PXD022881[https://www.ebi.ac.uk/pride/archive/projects/PXD022881], PXD003881[https://www.ebi.ac.uk/pride/archive/projects/PXD003881], PXD027546[https://www.ebi.ac.uk/pride/archive/projects/PXD027546], PXD025634[https://www.ebi.ac.uk/pride/archive/projects/PXD025634], PXD002838[https://www.ebi.ac.uk/pride/archive/projects/PXD002838] and PXD002882[https://www.ebi.ac.uk/pride/archive/projects/PXD002882], respectively.
Datasets NCC19, SM1100, MM, SO and GUS can be downloaded from the MetaboLights database under the accession numbers MTBLS1866[https://www.ebi.ac.uk/metabolights/editor/MTBLS1866], MTBLS733[https://www.ebi.ac.uk/metabolights/editor/MTBLS733], MTBLS5430[https://www.ebi.ac.uk/metabolights/editor/MTBLS5430], MTBLS492[https://www.ebi.ac.uk/metabolights/editor/MTBLS492] and MTBLS650[https://www.ebi.ac.uk/metabolights/editor/MTBLS650], respectively.
Datasets HCC-R2, Benchmark-FC, Benchmark-QC-H, Benchmark-QC-E, Benchmark-RT and Benchmark-MV can be downloaded from the iProX database under the accession numbers IPX0006622000[https://www.iprox.cn//page/project.html?id=IPX0006622000], IPX0006638000[https://www.iprox.cn//page/project.html?id=IPX0006638000], IPX0006819000[https://www.iprox.cn//page/project.html?id=IPX0006819000], IPX0006819000[https://www.iprox.cn//page/project.html?id=IPX0006819000], IPX0006820000[https://www.iprox.cn//page/project.html?id=IPX0006820000] and IPX0007319000[https://www.iprox.cn//page/project.html?id=IPX0007319000], respectively.
All other relevant data supporting the key findings of this study are available within the article or the Supplementary Information files. Source data are provided with this paper.

## Research involving human participants, their data, or biological material

Policy information about studies with human participants or human data. See also policy information about sex, gender (identity/presentation), and sexual orientation and race, ethnicity and racism.

| | |
|---|---|
| Reporting on sex and gender | In this study, patients were randomized according to inclusion criteria and sex was not considered in study design. Sex was determined based on patient self-reporting. The sex information was collected from case report of each patient and written informed consents were obtained from all patients (N=23). Sex-based analyses were not involved in this study. Because HCC has a strong male predominance (male to female ratio of 2-3:1) as stated in an HCC review (Llovet, J.M., Kelley, R.K., Villanueva, A. et al. Hepatocellular carcinoma. Nat Rev Dis Primers 7, 6 (2021). https://doi.org/10.1038/s41572-020-00240-3). We did not enroll enough female HCC patients. |
| Reporting on race, ethnicity, or other socially relevant groupings | All patients (N=23) in dataset HCC-R2 were Chinese. We did not include socially relevant categorical variables except for sex and age. HCC-R2 includes 22 male and 1 female. 14 patients were younger than 60 years old and 9 patients were over 60 years old when they underwent the surgery. |
| Population characteristics | See above. |
| Recruitment | HCC tissues were obtained from patients underwent a curative-intent liver resection at the Eastern Hepatobiliary Surgery Hospital in Shanghai between 2012 and 2014.<br>All patients met the following eligibility criteria: had a histopathologically-confirmed hepatocellular carcinoma; underwent a curative-intent liver resection that was defined as complete removal of macroscopic tumor nodules with microscopic tumor-free resection margins, and without macroscopic vascular invasion or extrahepatic metastasis; had tumors at stage 0, A or B according to the Barcelona Clinic Liver Cancer (BCLC) staging system (European Association for the Study of the Liver, 2018); had no preoperative anti-cancer therapy; did not die during the perioperative period; had no disease residue on imaging studies within two months of surgery; did not participate in any clinical trials before disease recurrence; and had complete clinicopathological and prognostic data. |
| Ethics oversight | Written informed consent was obtained from all patients. The study was approved by the ethics committee of the Eastern Hepatobiliary Surgery Hospital in Shanghai. |

Note that full information on the approval of the study protocol must also be provided in the manuscript.

# Field-specific reporting

Please select the one below that is the best fit for your research. If you are not sure, read the appropriate sections before making your selection.

☒ Life sciences ☐ Behavioural & social sciences ☐ Ecological, evolutionary & environmental sciences

For a reference copy of the document with all sections, see nature.com/documents/nr-reporting-summary-flat.pdf

# Life sciences study design

All studies must disclose on these points even when the disclosure is negative.

| | |
|---|---|
| Sample size | The HCC datasets in this study were from observational studies. Sample size was not predetermined. |
| Data exclusions | No data exclusions are involved. |
| Replication | To evaluate and validate the reproducibility of the RT alignment algorithm DeepRTAlign considering technical or biological variances, some datasets had technical or biological repeats using one LC-MS instruments or several different instruments. All attempts at replication were successful. |

| | |
|---|---|
| Randomization | No randomization of samples is involved. Because the HCC-R2 study is observational and this work focuses on a novel RT alignment algorithm (DeepRTAlign). The datasets used in this study were only used to train and validate the algorithm. |
| Blinding | No blinding is involved. Because the HCC-R2 study is observational and this work focuses on a novel RT alignment algorithm (DeepRTAlign). The datasets used in this study were only used to train and validate the algorithm. |

# Reporting for specific materials, systems and methods

We require information from authors about some types of materials, experimental systems and methods used in many studies. Here, indicate whether each material, system or method listed is relevant to your study. If you are not sure if a list item applies to your research, read the appropriate section before selecting a response.

## Materials & experimental systems

| n/a | Involved in the study | |
|---|---|---|
| ☒ | ☐ | Antibodies |
| ☐ | ☒ | Eukaryotic cell lines |
| ☒ | ☐ | Palaeontology and archaeology |
| ☒ | ☐ | Animals and other organisms |
| ☒ | ☐ | Clinical data |
| ☒ | ☐ | Dual use research of concern |
| ☒ | ☐ | Plants |

## Methods

| n/a | Involved in the study | |
|---|---|---|
| ☒ | ☐ | ChIP-seq |
| ☒ | ☐ | Flow cytometry |
| ☒ | ☐ | MRI-based neuroimaging |

## Eukaryotic cell lines

Policy information about cell lines and Sex and Gender in Research

| | |
|---|---|
| Cell line source(s) | HEK 293T cell line was obtained from National Infrastructure Cell Line Resource (Beijing, China) . |
| Authentication | Using short tandem repeat (STR) profiling method. |
| Mycoplasma contamination | HEK 293T cell line was tested for mycoplasma contamination every month. The mycoplasma contamination test results were negative. |
| Commonly misidentified lines (See ICLAC register) | No commonly misidentified cell lines were used. |

## Plants

| | |
|---|---|
| Seed stocks | NA |
| Novel plant genotypes | NA |
| Authentication | NA |
