# Supplementary material for: DeepRTAlign: toward accurate retention time alignment for large cohort mass spectrometry data analysis
Source: Nat Commun. 2023 Dec 11;14:8188. doi: 10.1038/s41467-023-43909-5 (PMC10713976; doi:10.1038/s41467-023-43909-5)
Supplement: Supplementary file 3 — Description of Additional Supplementary Files [file 41467_2023_43909_MOESM3_ESM.pdf]

Title: Supplementary Data 1.
Description: An Excel file that contains the 15 aligned MS1 features in the feature-based early-recurrence classifier for HCC early recurrence prediction. Their identification results from both DDA data and sPRM data were provided.

Title: Supplementary Data 2.
Description: A csv file that contains grouping information of the dataset HCC-R2 (N=23).

Title: Supplementary Data 3.
Description: The mass list of target precursors for PRM experiment. A total of 49 features including the 15 features and other 34 features, were selected from the top 200 MS features in dataset HCC-T, as the targeted precursors in the mass list table in the PRM experiment. 11 iRT standards (iRT kit, Biognosys) was included in the mass list for retention time calibration in Skyline analysis. For four features, precursors with two different charges were included in the mass list. Therefore, a total of 64 targeted precursors were set in the mass list. Targeted precursors were isolated through a window of 1.2 Th. Normalized collision energy was set to 28%.

Title: Supplementary Data 4.
Description: The peptide identification results of PD 2.5 for the PRM data of HCC-R2 cohort (N=23). Trypsin was selected as the proteolytic enzyme, and missed cleavage sites were allowed up to two. Cysteine carbamidomethylation was set as the static modification. The oxidation of methionine and the acetylation of the protein N-terminal were set as the dynamic modifications. The precursor mass tolerance was 10 ppm, and the fragment mass tolerance was 0.02 Da. The false discovery rates of the peptide-spectrum matches (PSMs) and proteins were set to less than 1%.

Title: Supplementary Data 5
Description: A document that contains details of the Skyline annotated spectra for the verified 48 features using PRM.

Title: Supplementary Data 6.
Description: Quantification results of the verified 48 features exported from Skyline, including precursors and transitions. Peak areas of precursors and transitions were summed as peptide abundance of the 15 features and used for the prediction of early recurrence.

Title: Supplementary Data 7.
Description: Parameters optimization using grid search for the RF model based on the 10-fold cross validation results of the training set HCC-T.

Title: Supplementary Data 8.
Description: Parameters optimization using grid search for the SVM model based on the 10-fold cross validation results of the training set HCC-T. All the other parameters were kept default in scikit-learn v0.21.3.
