# Supplementary material for: DeepRTAlign: toward accurate retention time alignment for large cohort mass spectrometry data analysis
Source: Nat Commun. 2023 Dec 11;14:8188. doi: 10.1038/s41467-023-43909-5 (PMC10713976; doi:10.1038/s41467-023-43909-5)
Supplement: Supplementary file 9 — Supplementary Data 5 [file 41467_2023_43909_MOESM9_ESM.docx]

**The 15 features:**


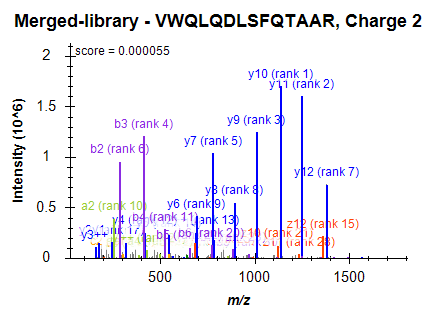


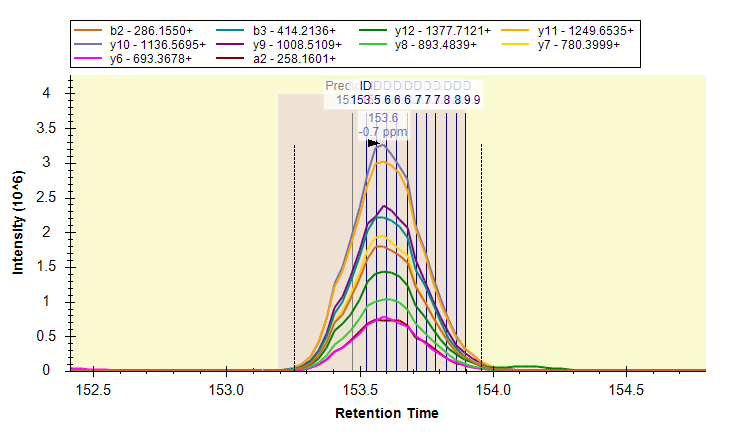


Annotated MS/MS spectrum for VWQLQDLSFQTAAR, matched for MS feature of doubly charged “1661.84_1661.87_12”.


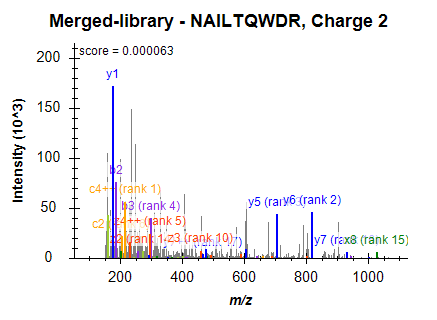


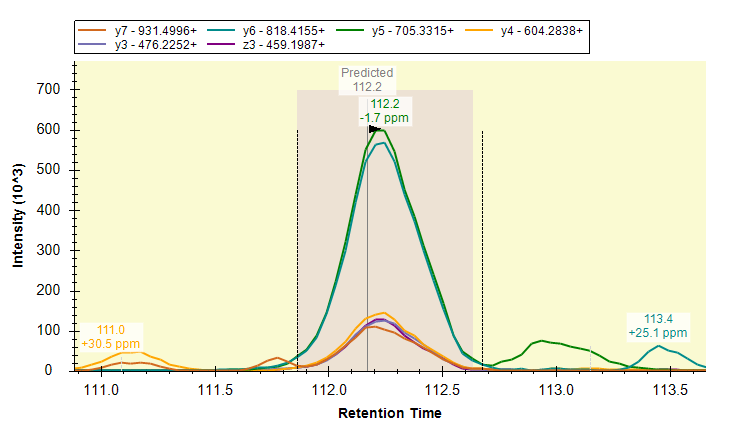


Annotated MS/MS spectrum for NAILTQWDR, matched for MS feature of doubly charged “1115.57_1115.6_23”.


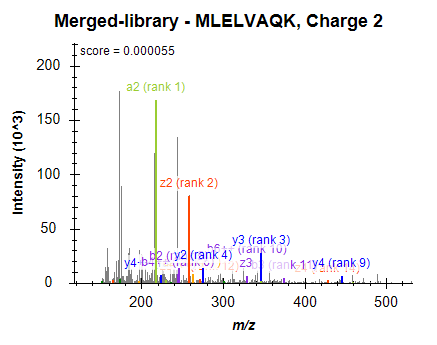


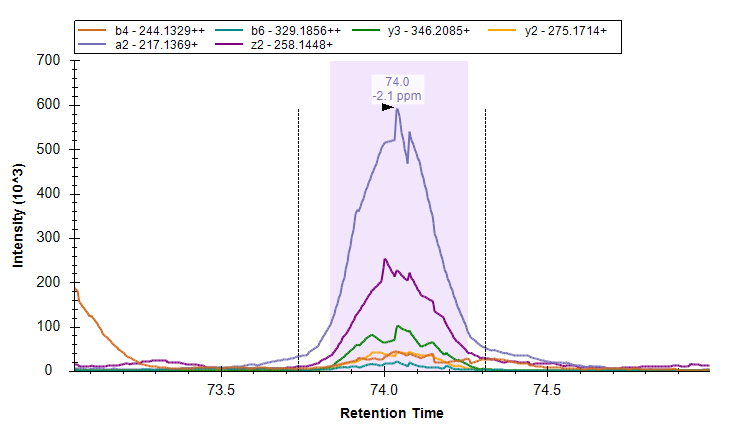


Annotated MS/MS spectrum for MLELVAQK, matched for MS feature of doubly charged “930.51_930.54_28”.


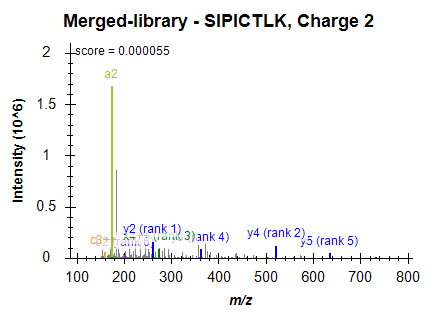


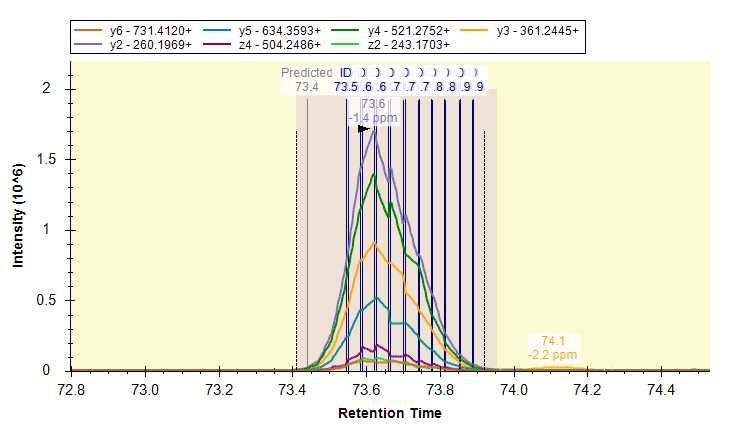


Annotated MS/MS spectrum for SIPIC[+57.021464]TLK, matched for MS feature of doubly charged “930.51_930.54_19”.


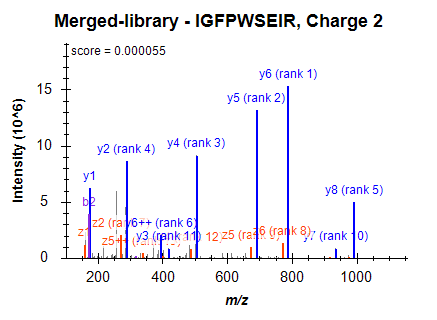


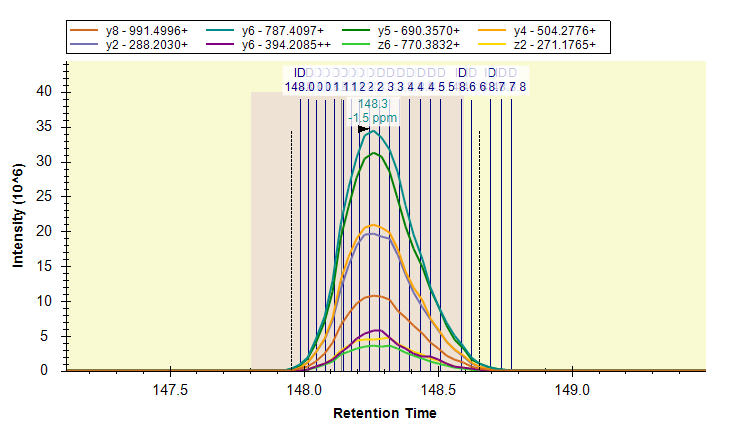


Annotated MS/MS spectrum for IGFPWSEIR, matched for MS feature of doubly charged “1103.57_1103.6_16”.


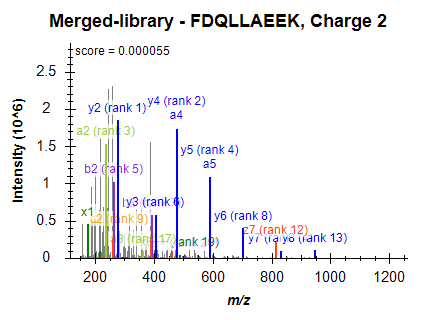


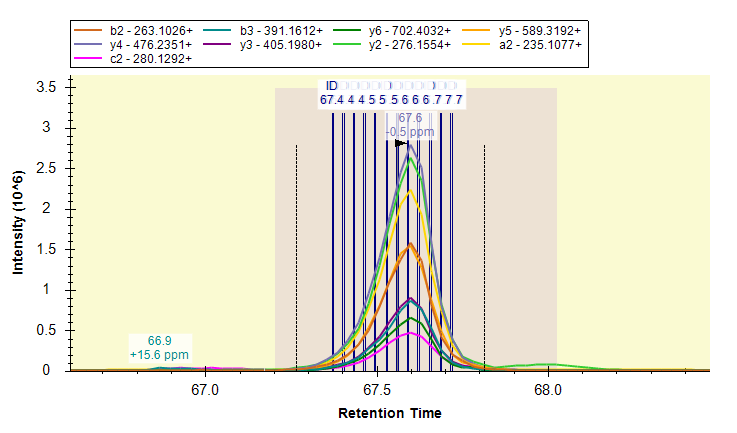


Annotated MS/MS spectrum for FDQLLAEEK, matched for MS feature of doubly charged “1091.54_1091.57_27”.


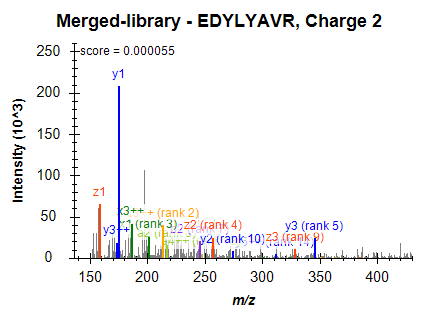


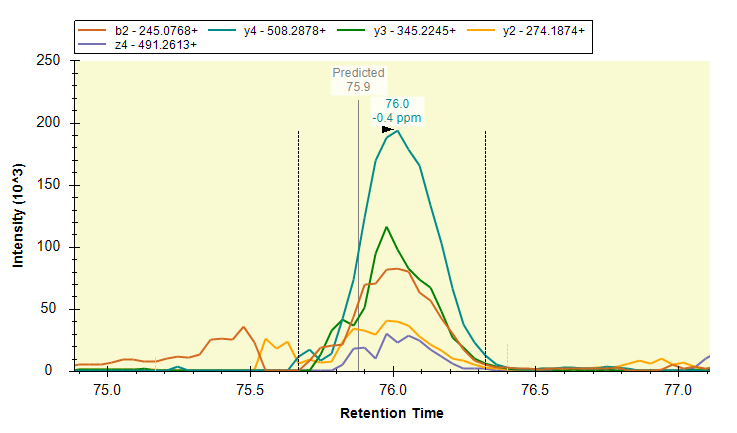


Annotated MS/MS spectrum for EDYLYAVR, matched for MS feature of doubly charged “1027.5_1027.53_53”.


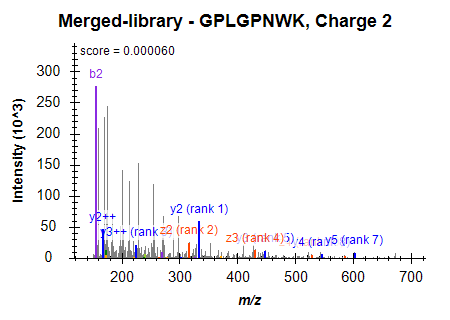


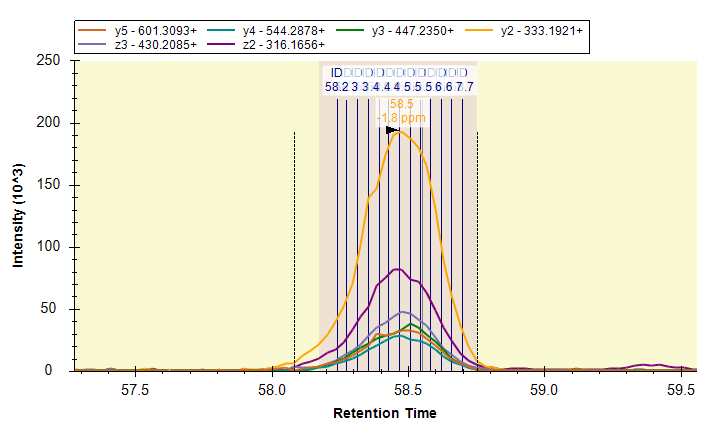


Annotated MS/MS spectrum for GPLGPNWK, matched for MS feature of doubly charged “867.45_867.48_4”.


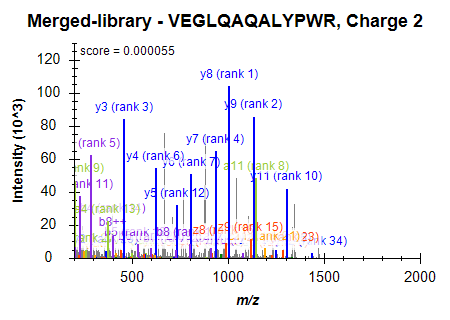


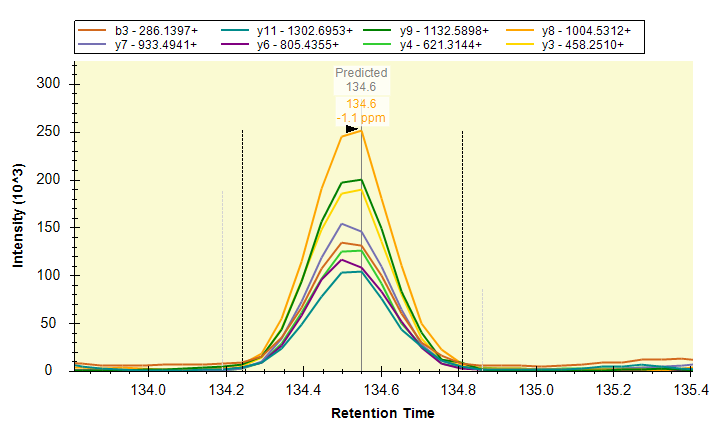


Annotated MS/MS spectrum for VEGLQAQALYPWR, matched for MS feature of doubly charged “1529.78_1529.81_2”.


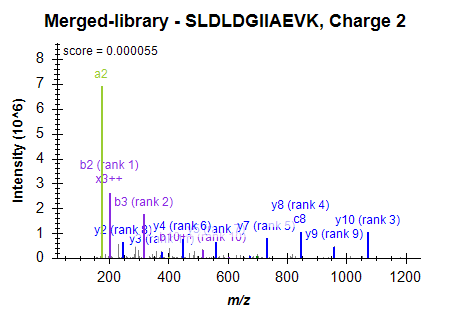


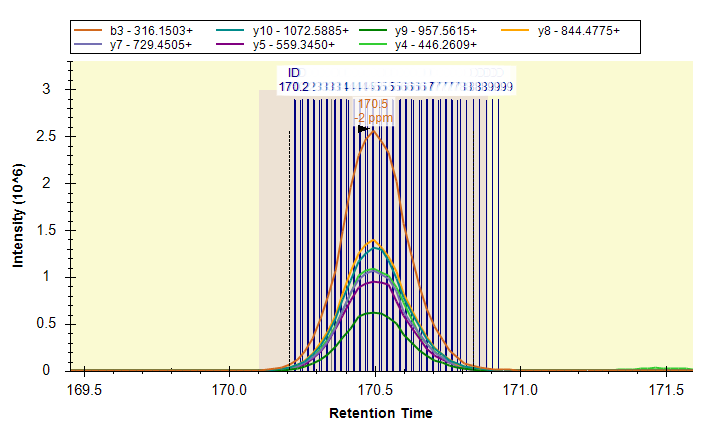


Annotated MS/MS spectrum for SLDLDGIIAEVK, matched for MS feature of doubly charged “1271.69_1271.72_5”.


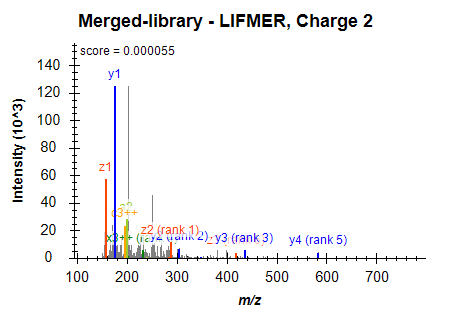


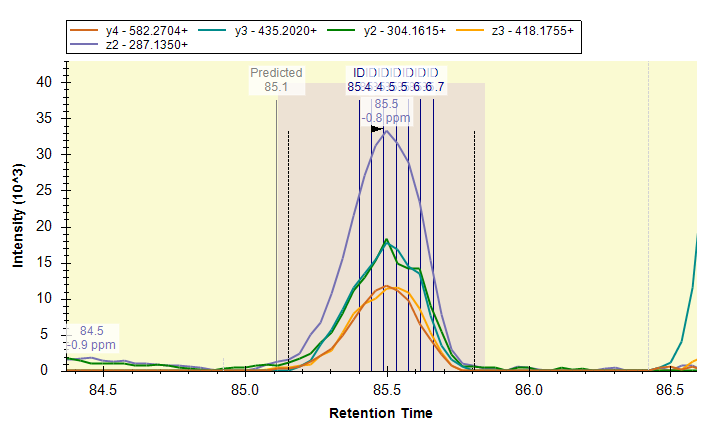


Annotated MS/MS spectrum for LIFMER, matched for MS feature of doubly charged “807.42_807.45_11”.


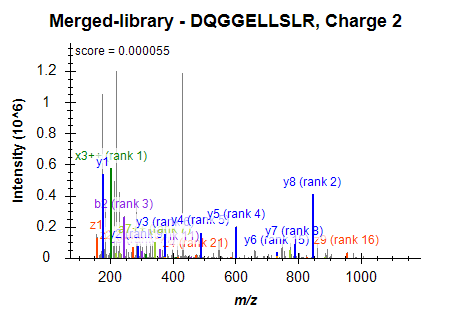


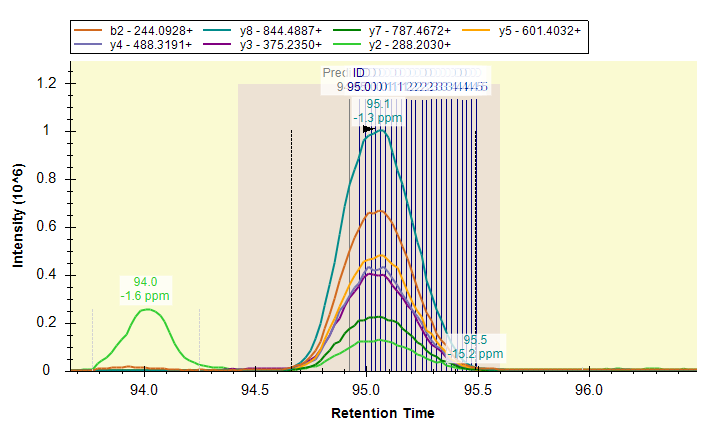


Annotated MS/MS spectrum for DQGGELLSLR, matched for MS feature of doubly charged “1086.56_1086.59_54”.


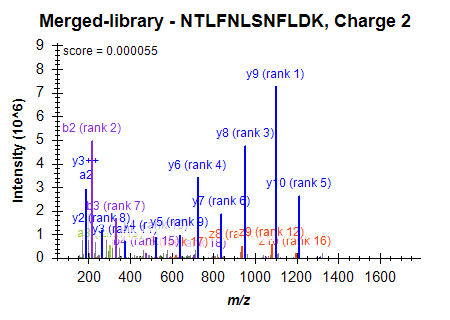


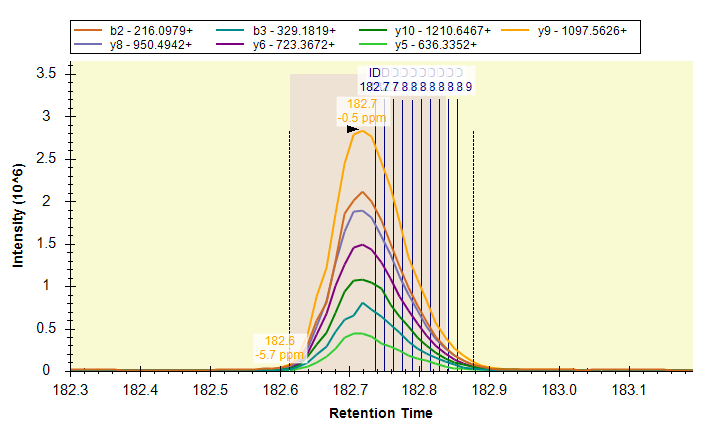


Annotated MS/MS spectrum for NTLFNLSNFLDK, matched for MS feature of doubly charged “1424.72_1424.75_33”.


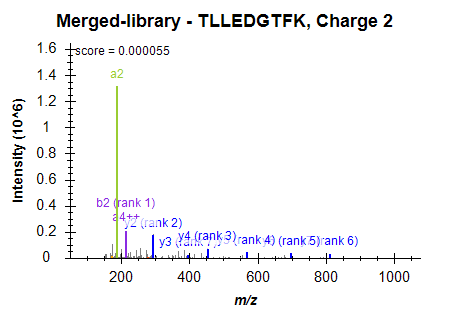


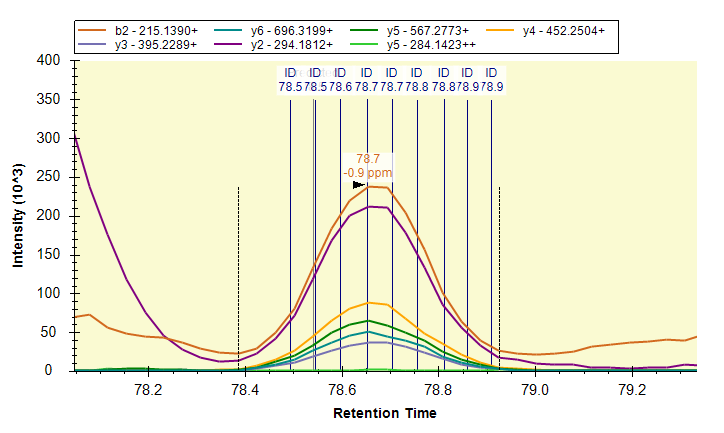
Annotated MS/MS spectrum for TLLEDGTFK, matched for MS feature of doubly charged “1022.52_1022.55_1”.


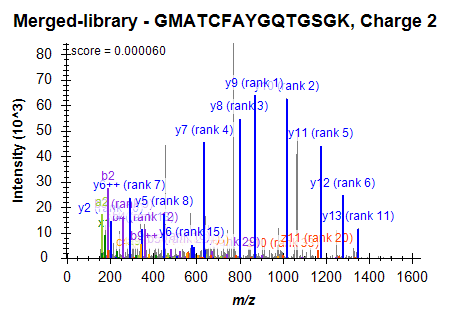


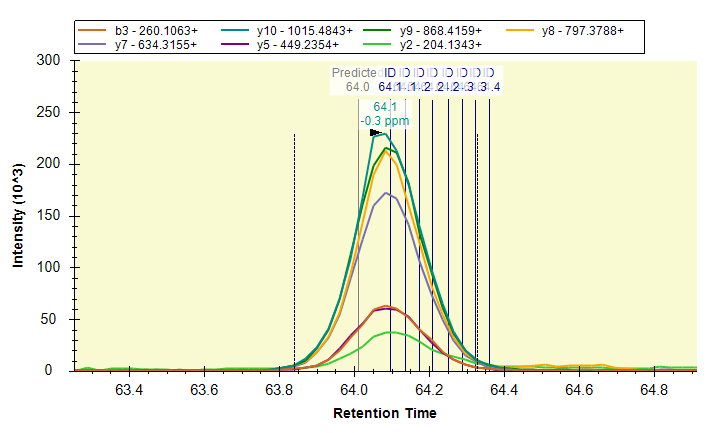


Annotated MS/MS spectrum for GMATC[+57.021464]FAYGQTGSGK, matched for MS feature of doubly charged “1534.64_1534.67_4”.

**The other 34 features:**


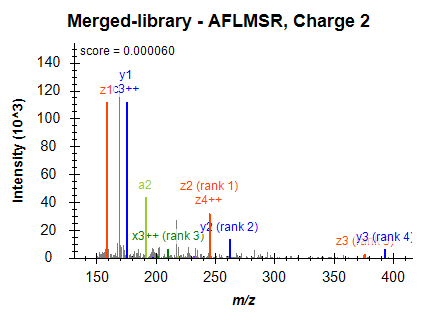


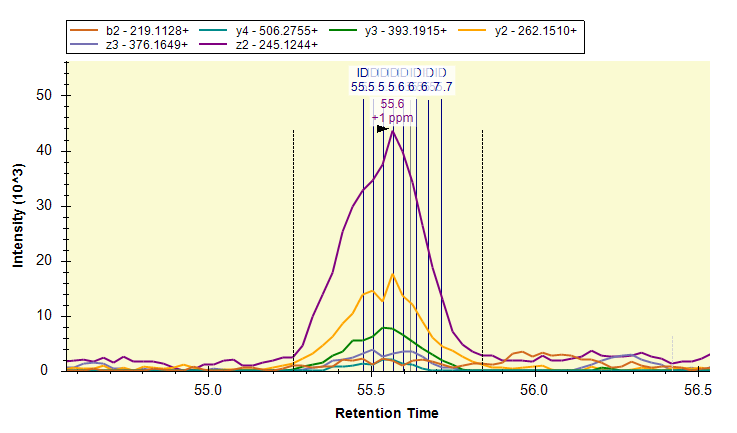


Annotated MS/MS spectrum for AFLMSR, matched for MS feature of doubly charged “723.36_723.39_8”.


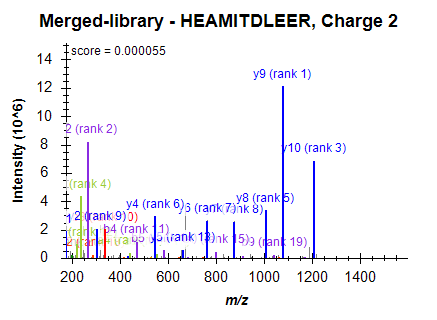


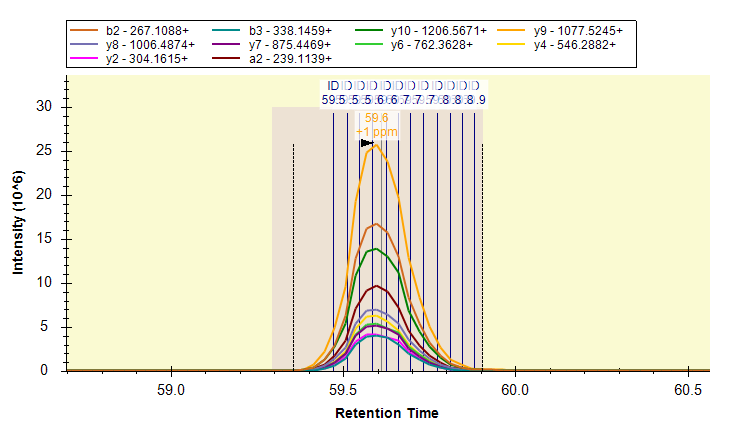


Annotated MS/MS spectrum for HEAMITDLEER, matched for MS feature of doubly charged “1342.61_1342.64_19”.


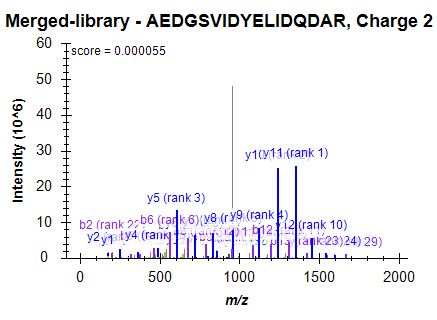


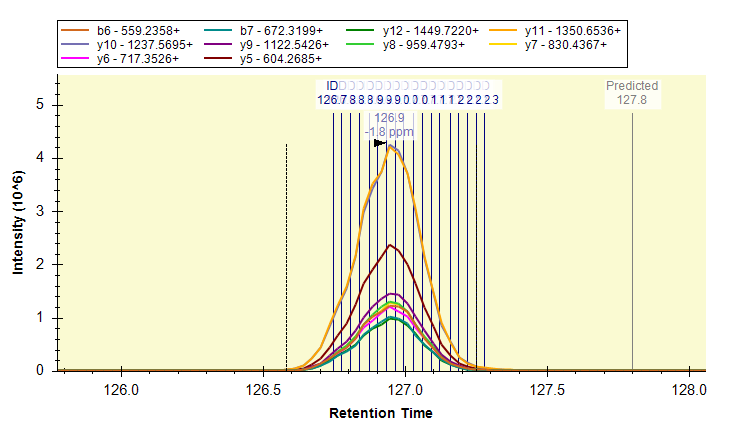


Annotated MS/MS spectrum for AEDGSVIDYELIDQDAR, matched for MS feature of doubly charged “1907.87_1907.9_3”.


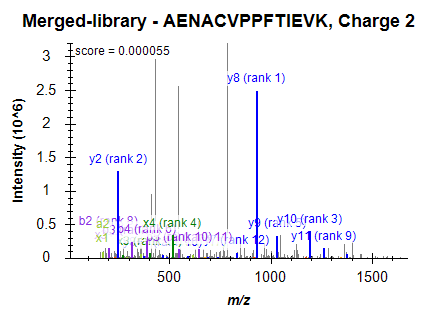


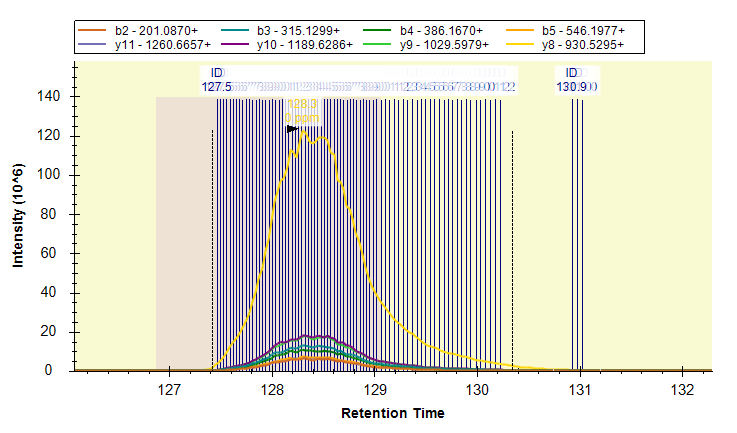


Annotated MS/MS spectrum for AENAC[+57.021464]VPPFTIEVK, matched for MS feature of doubly charged “1573.77_1573.8_2”.


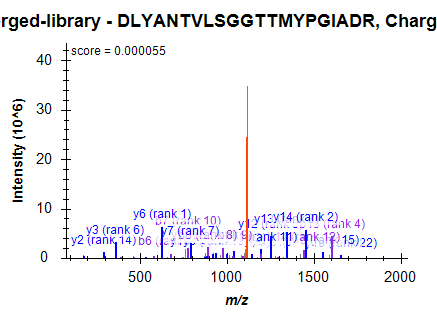


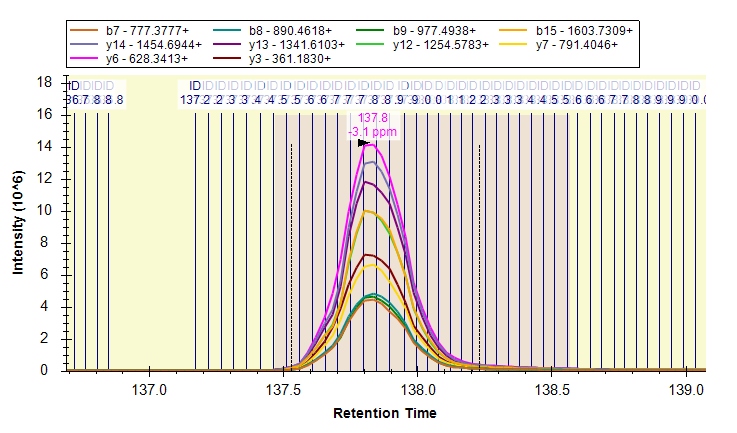


Annotated MS/MS spectrum for doubly charged DLYANTVLSGGTTM[+15.994915]YPGIADR, matched for MS feature of “2230.05_2230.08_40”.


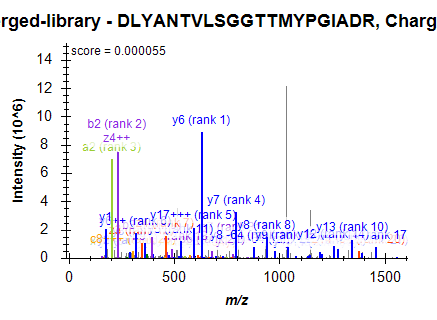


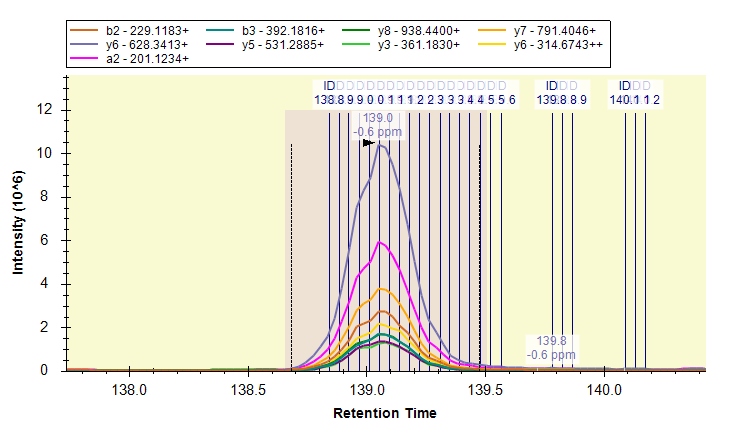


Annotated MS/MS spectrum for triply charged DLYANTVLSGGTTM[+15.994915]YPGIADR, matched for MS feature of “2230.05_2230.08_40”.


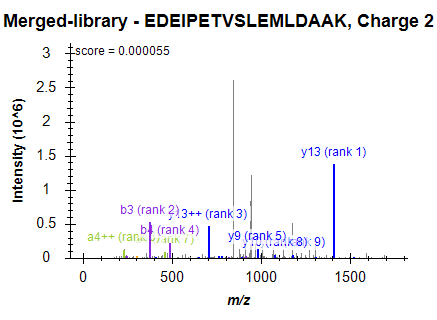


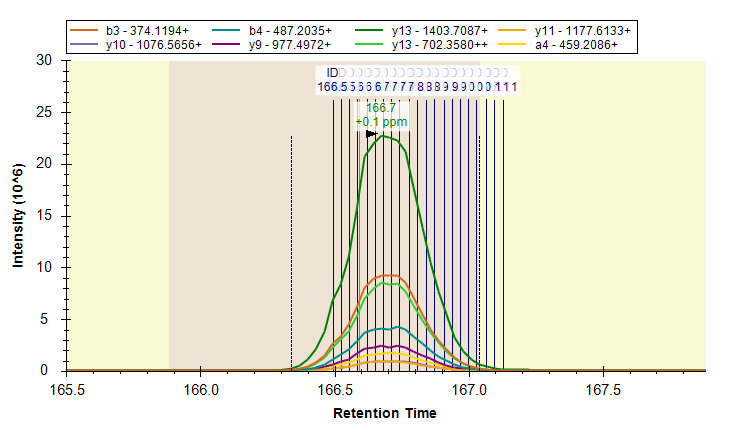


Annotated MS/MS spectrum for triply charged EDEIPETVSLEMLDAAK, matched for MS feature of doubly charged “1888.89_1888.92_1”.


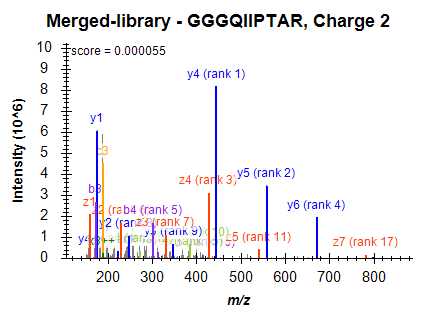


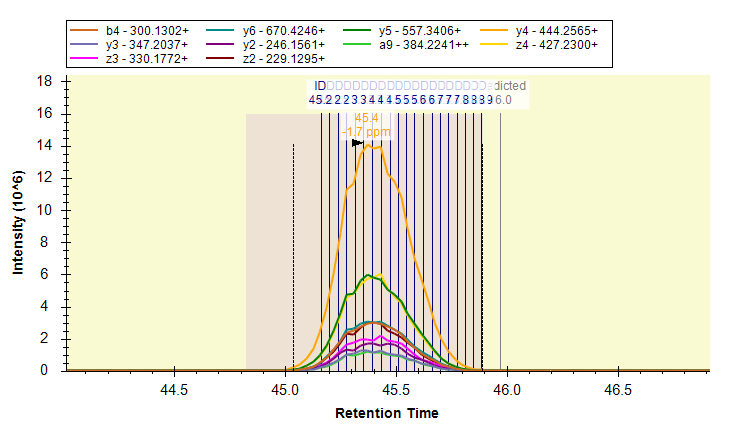


Annotated MS/MS spectrum for triply charged GGGQIIPTAR, matched for MS feature of doubly charged “968.53_968.56_1”.


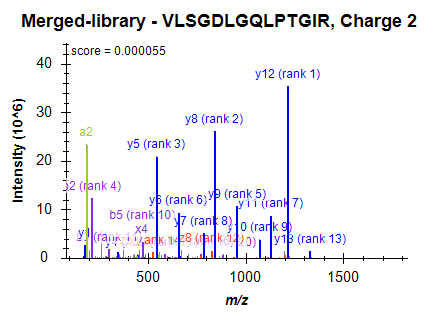


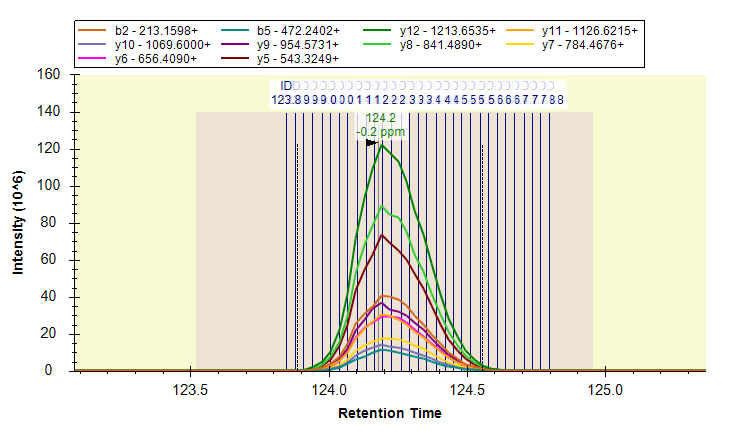


Annotated MS/MS spectrum for triply charged VLSGDLGQLPTGIR, matched for MS feature of doubly charged “1424.79_1424.82_2”.


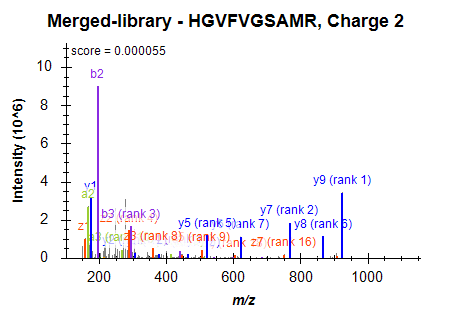


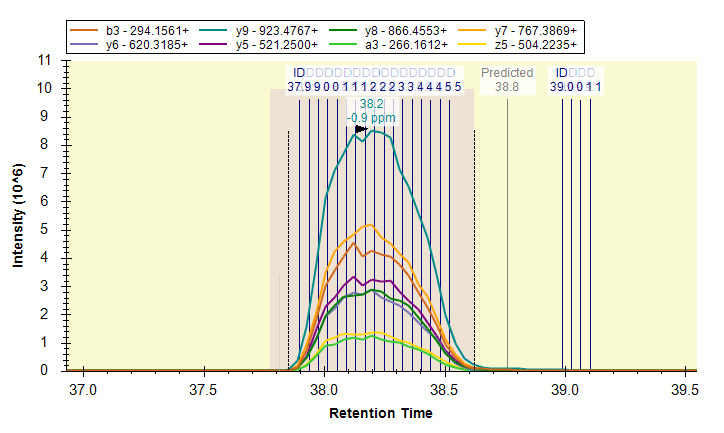


Annotated MS/MS spectrum for triply charged HGVFVGSAMR, matched for MS feature of doubly charged “1059.52_1059.55_5”.


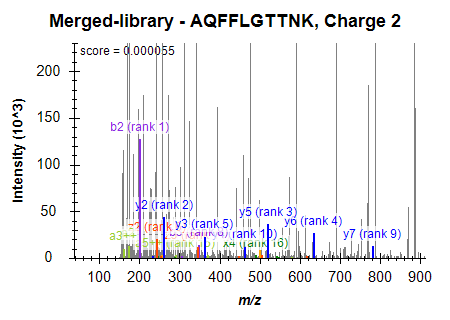


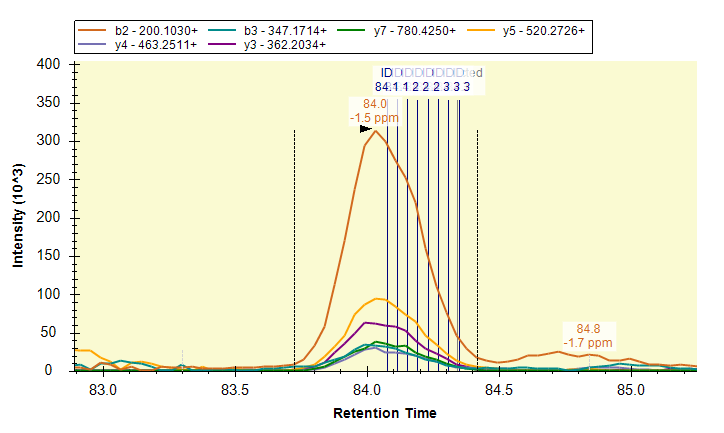


Annotated MS/MS spectrum for triply charged AQFFLGTTNK, matched for MS feature of doubly charged “1125.57_1125.6_13”.


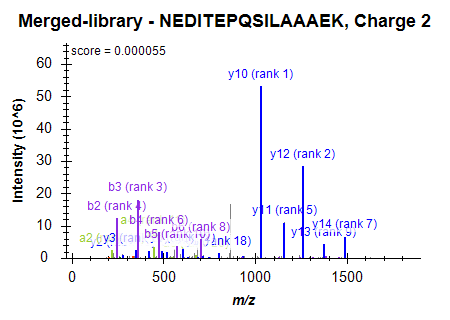


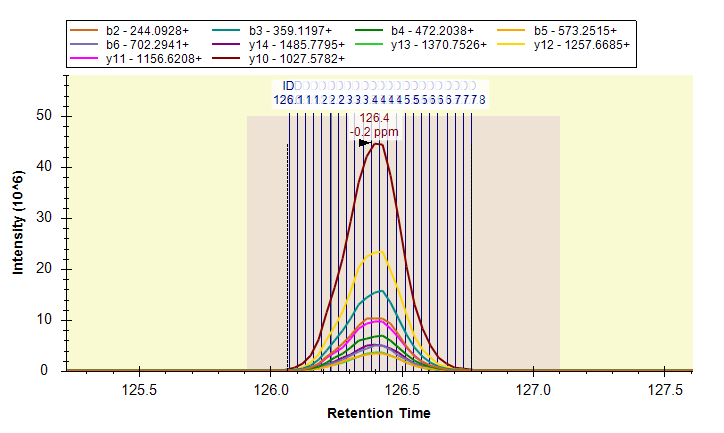


Annotated MS/MS spectrum for triply charged NEDITEPQSILAAAEK, matched for MS feature of doubly charged “1727.85_1727.88_0”.


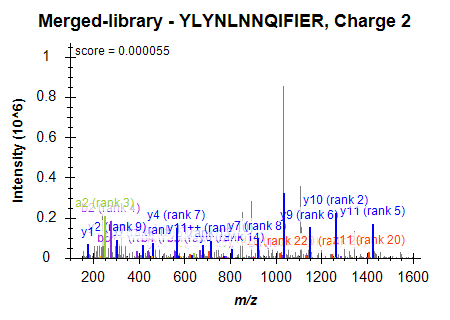


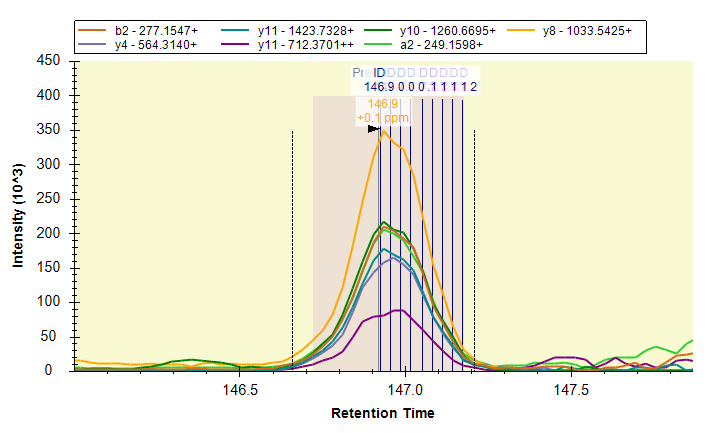


Annotated MS/MS spectrum for triply charged YLYNLNNQIFIER, matched for MS feature of doubly charged “1698.86_1698.89_2”.


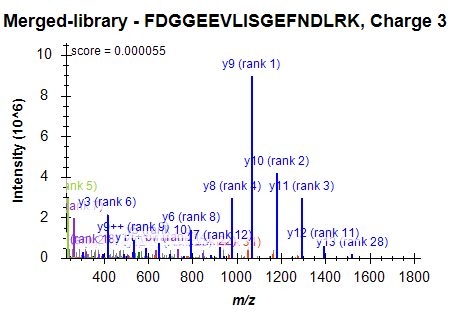

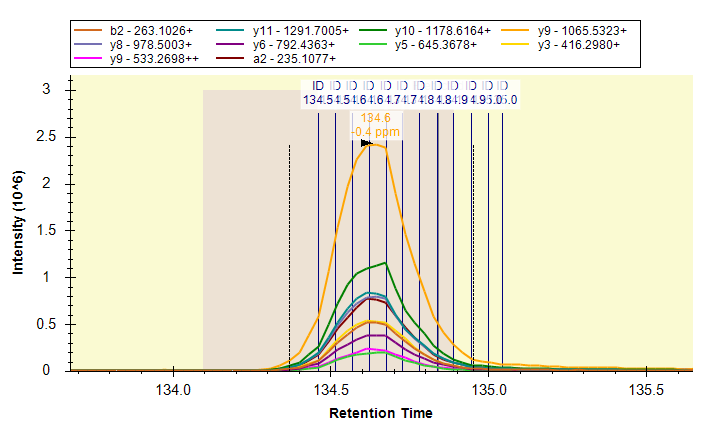


Annotated MS/MS spectrum for triply charged FDGGEEVLISGEFNDLRK, matched for MS feature of triply charged “2023.98_2024.01_3”.


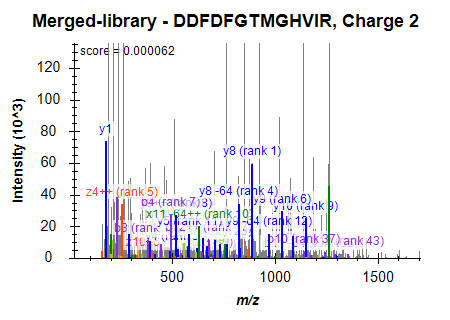


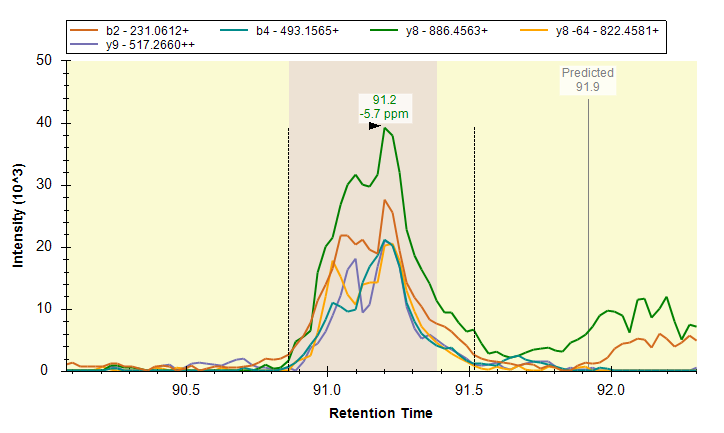


Annotated MS/MS spectrum for doubly charged DDFDFGTM[+15.994915]GHVIR, matched for MS feature of triply charged “2023.98_2024.01_3”.


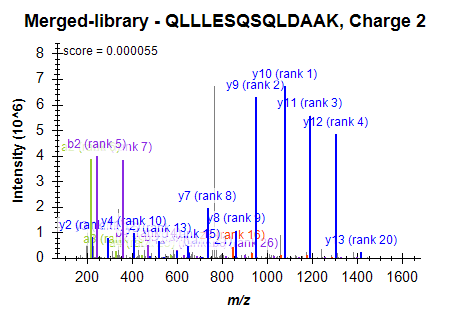


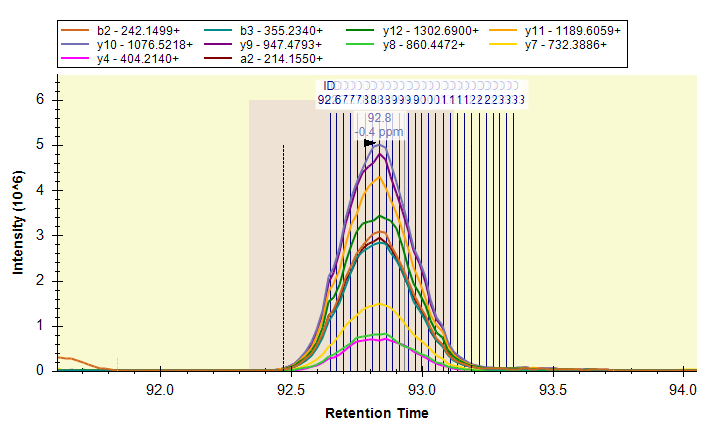


Annotated MS/MS spectrum for triply charged QLLLESQSQLDAAK, matched for MS feature of doubly charged “1542.81_1542.84_13”.


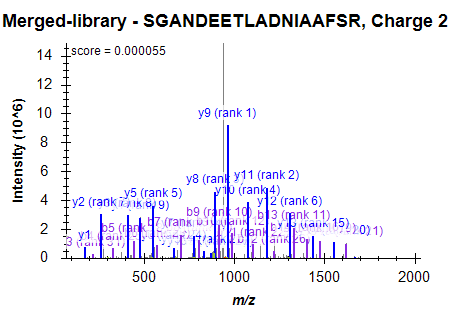


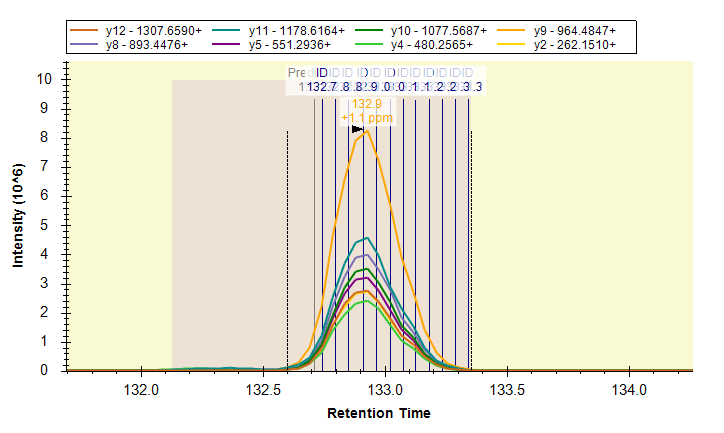


Annotated MS/MS spectrum for doubly charged SGANDEETLADNIAAFSR, matched for MS feature of doubly charged “1879.85_1879.88_0”.


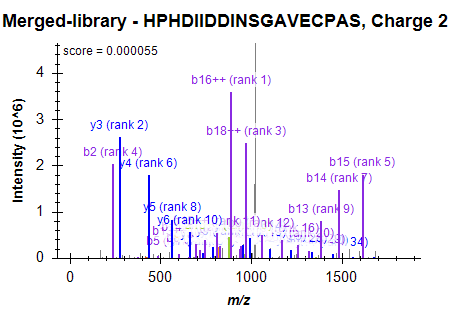


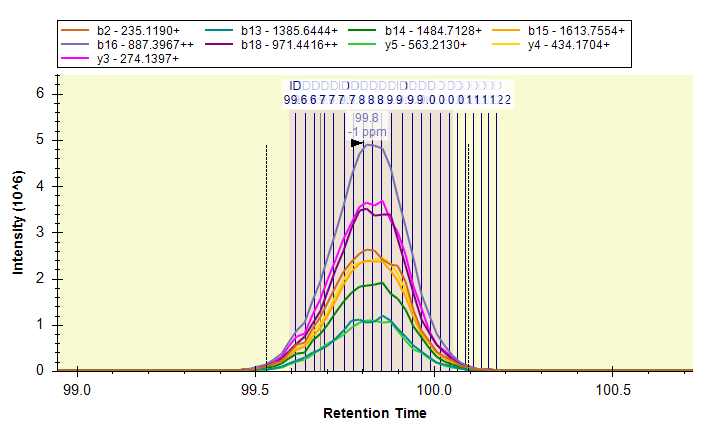


Annotated MS/MS spectrum for doubly charged HPHDIIDDINSGAVEC[+57.021464]PAS, matched for MS feature of doubly charged “2045.9_2045.93_4”.


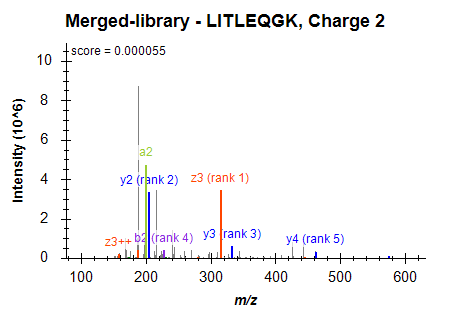


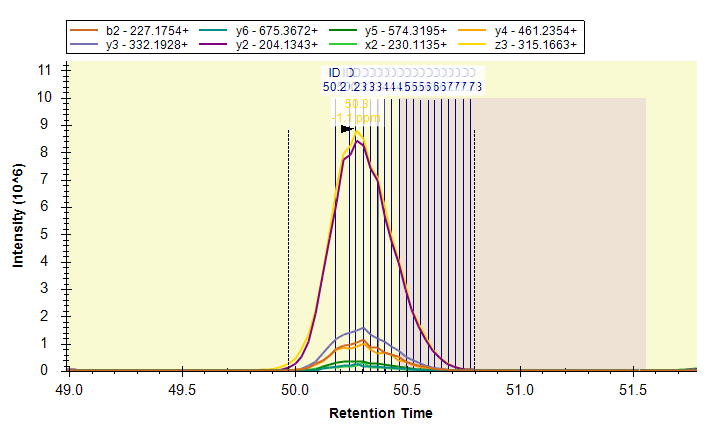


Annotated MS/MS spectrum for doubly charged LITLEQGK, matched for MS feature of doubly charged “900.52_900.55_9”.


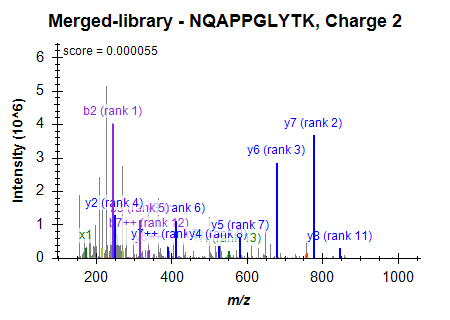


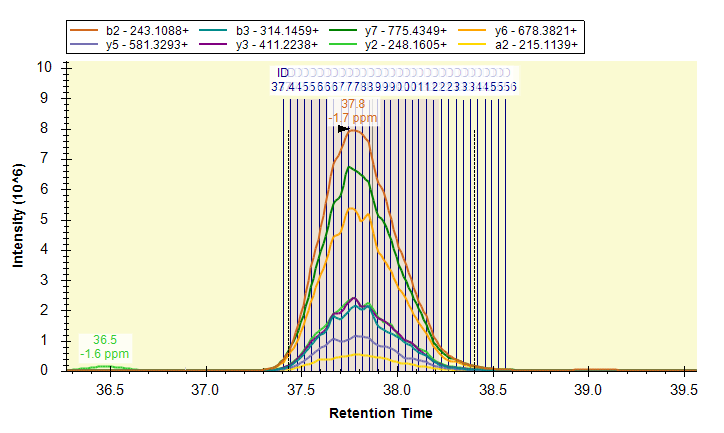


Annotated MS/MS spectrum for doubly charged NQAPPGLYTK, matched for MS feature of doubly charged “1087.56_1087.59_61”.


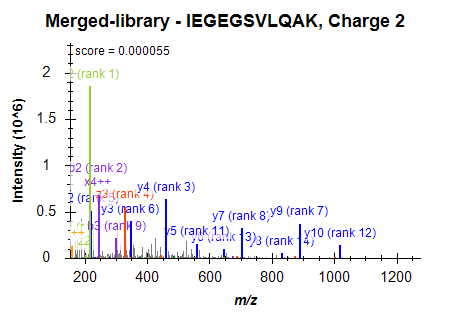


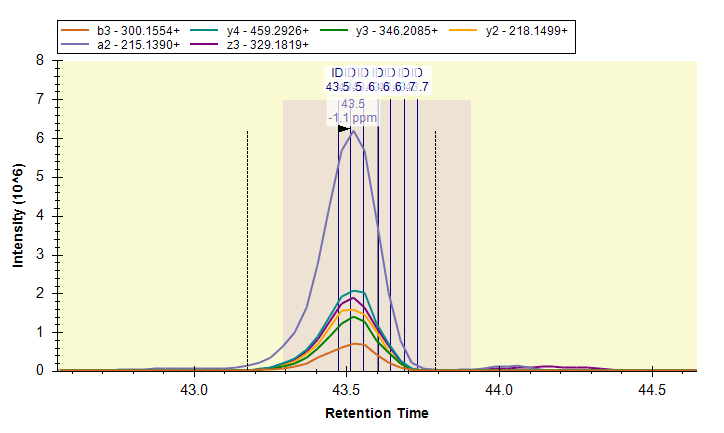


Annotated MS/MS spectrum for doubly charged IEGEGSVLQAK, matched for MS feature of doubly charged “1129.59_1129.62_7”.


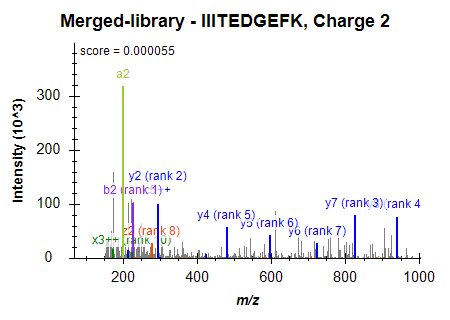


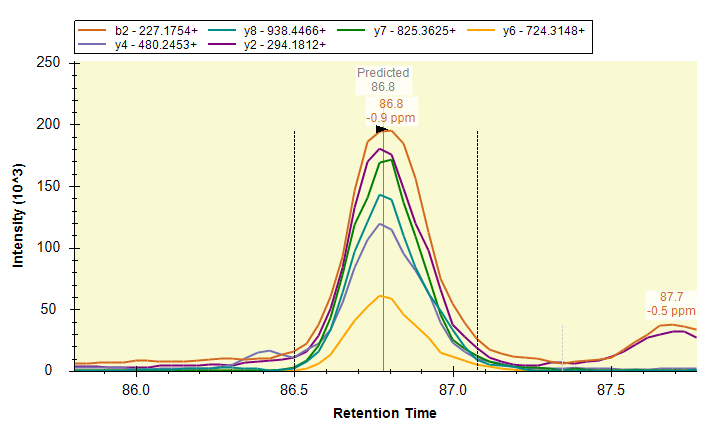


Annotated MS/MS spectrum for doubly charged IIITEDGEFK, matched for MS feature of doubly charged “1163.6_1163.63_10”.


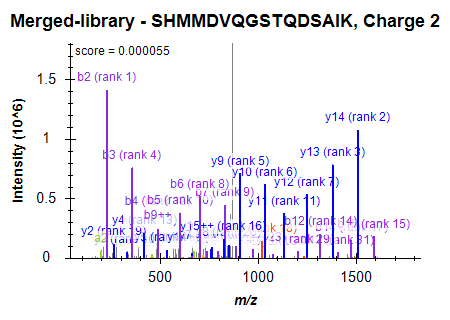


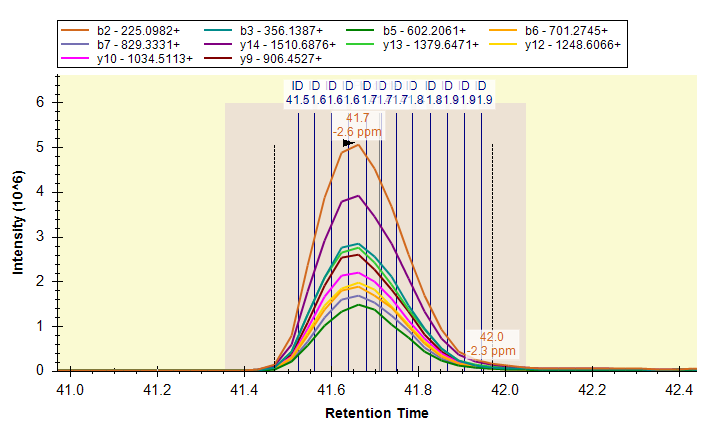


Annotated MS/MS spectrum for doubly charged SHMMDVQGSTQDSAIK, matched for MS feature of doubly charged “1733.76_1733.79_0”.


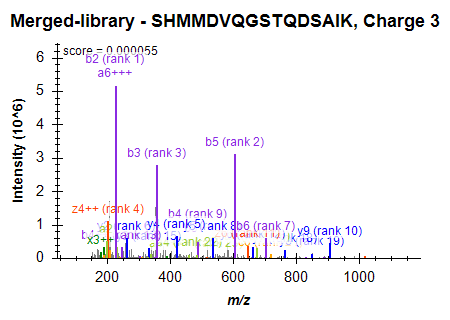


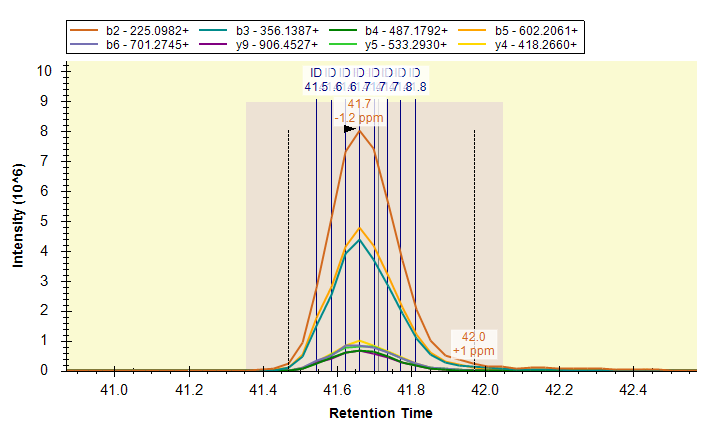


Annotated MS/MS spectrum for doubly charged SHMMDVQGSTQDSAIK, matched for MS feature of triply charged “1733.76_1733.79_0”.


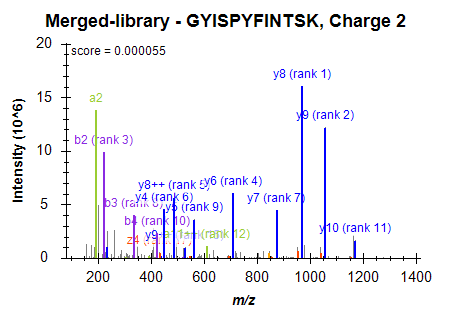


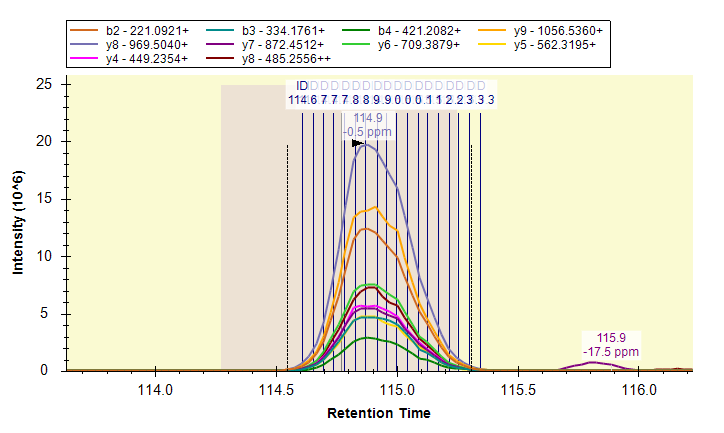


Annotated MS/MS spectrum for doubly charged GYISPYFINTSK, matched for MS feature of triply charged “1388.69_1388.72_30”.


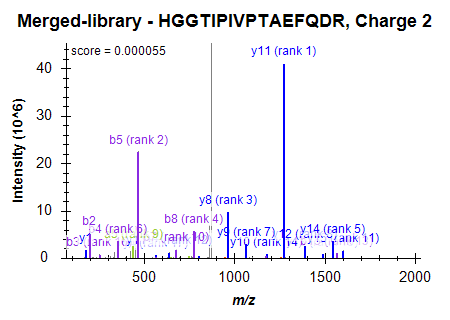


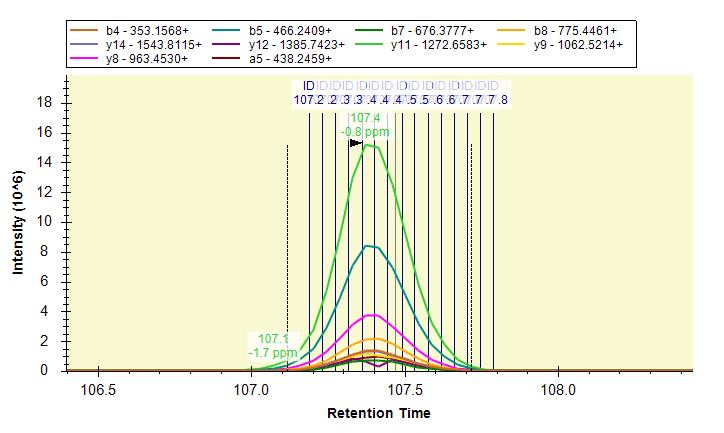


Annotated MS/MS spectrum for doubly charged HGGTIPIVPTAEFQDR, matched for MS feature of doubly charged “1736.88_1736.91_0”.


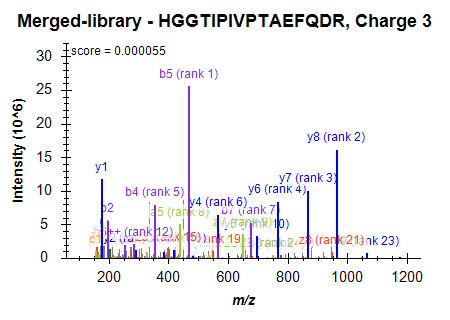

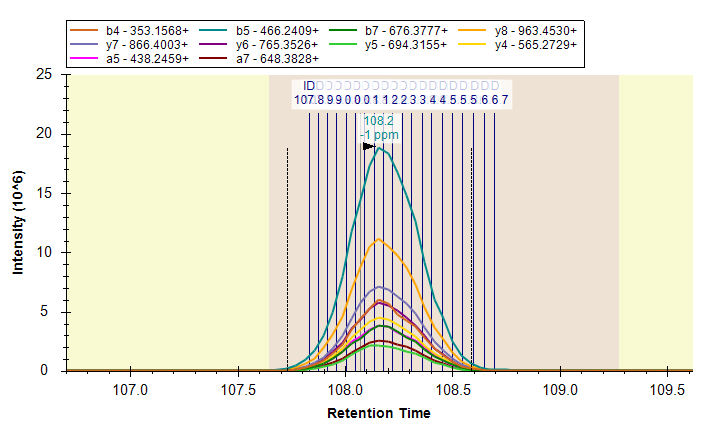


Annotated MS/MS spectrum for doubly charged HGGTIPIVPTAEFQDR, matched for MS feature of triply charged “1736.88_1736.91_0”.


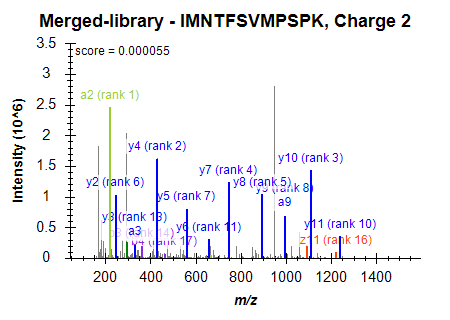


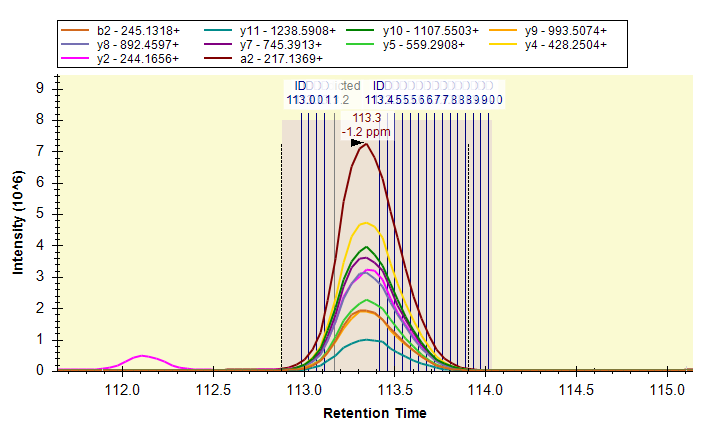


Annotated MS/MS spectrum for doubly charged IMNTFSVMPSPK, matched for MS feature of doubly charged “1350.66_1350.69_11”.


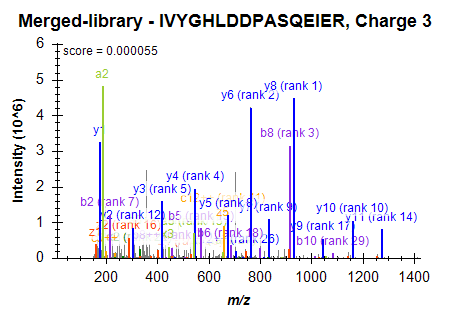


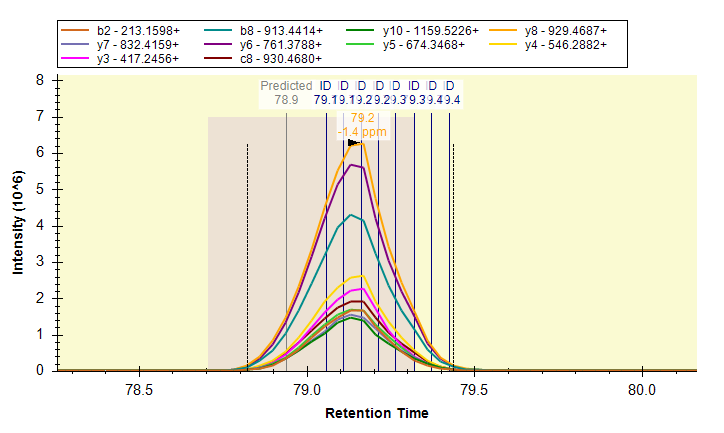


Annotated MS/MS spectrum for doubly charged IVYGHLDDPASQEIER, matched for MS feature of triply charged “1840.89_1840.92_8”.


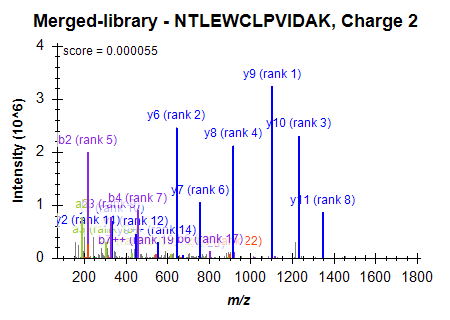

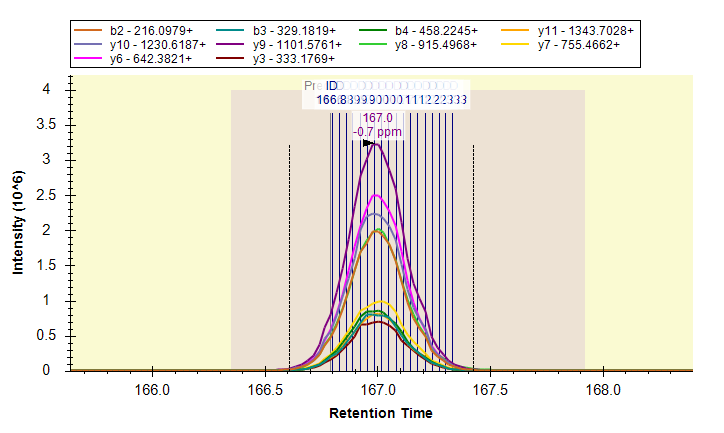


Annotated MS/MS spectrum for doubly charged NTLEWC[+57.021464]LPVIDAK, matched for MS feature of doubly charged “1557.77_1557.8_3”.


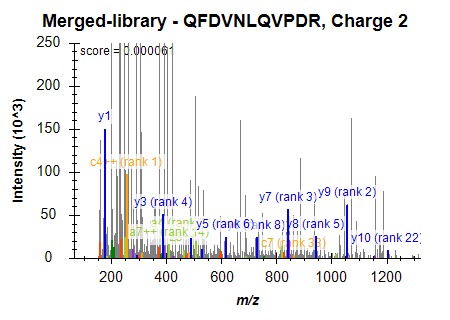


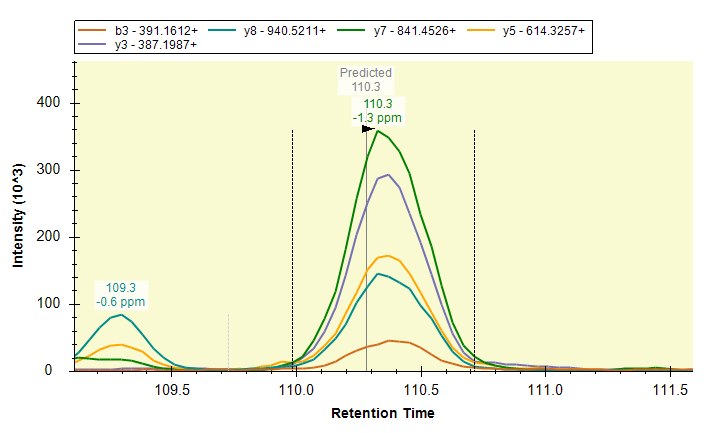


Annotated MS/MS spectrum for doubly charged QFDVNLQVPDR, matched for MS feature of doubly charged “1329.66_1329.69_17”.


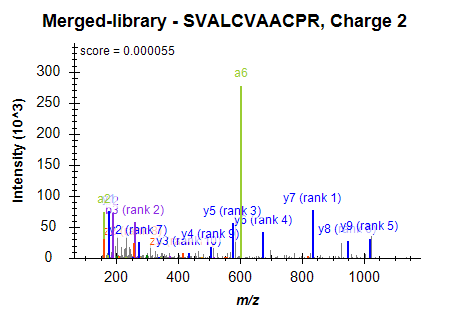


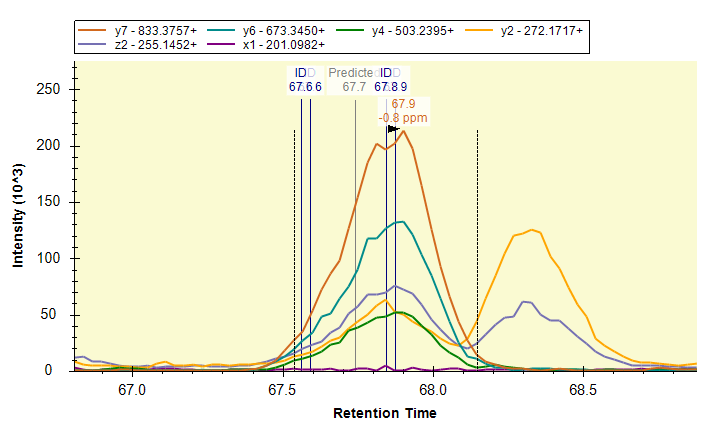


Annotated MS/MS spectrum for doubly charged SVALC[+57.021464]VAAC[+57.021464]PR, matched for MS feature of doubly charged “1202.58_1202.61_17 ”.


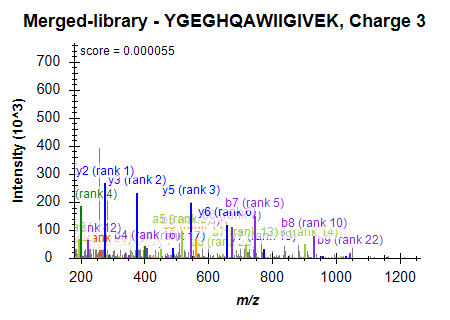

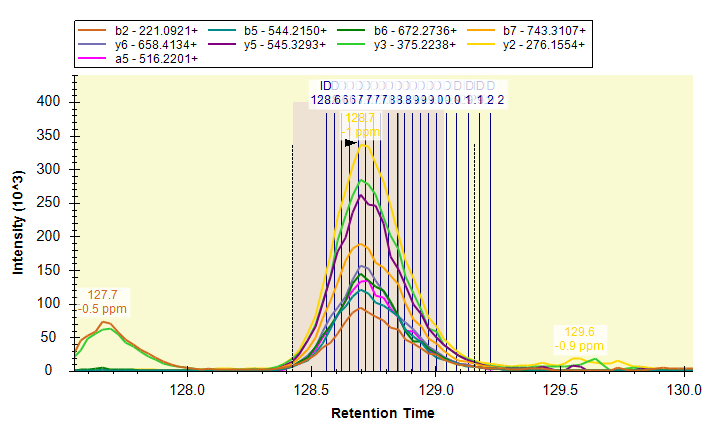


Annotated MS/MS spectrum for doubly charged YGEGHQAWIIGIVEK, matched for MS feature of triply charged “1698.86_1698.89_2”.


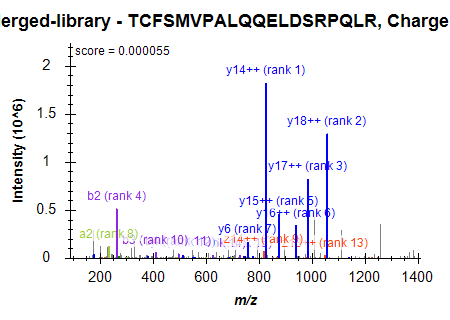


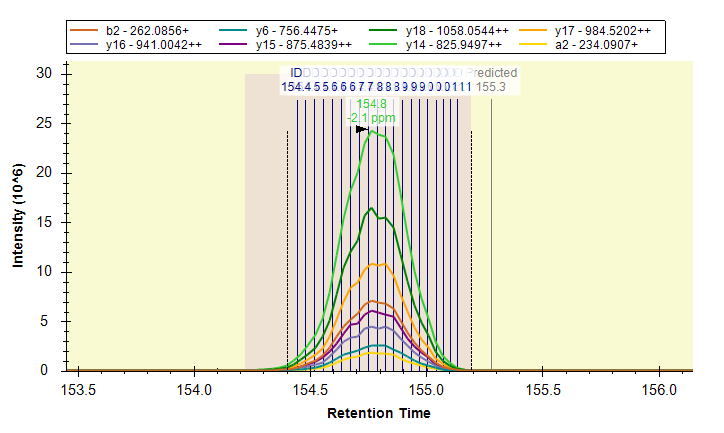


Annotated MS/MS spectrum for doubly charged TC[+57.021464]FSMVPALQQELDSRPQLR, matched for MS feature of triply charged “2375.16_2375.19_6”.


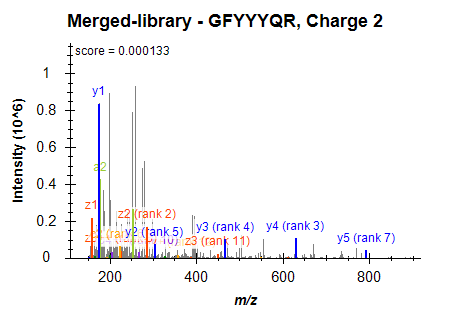

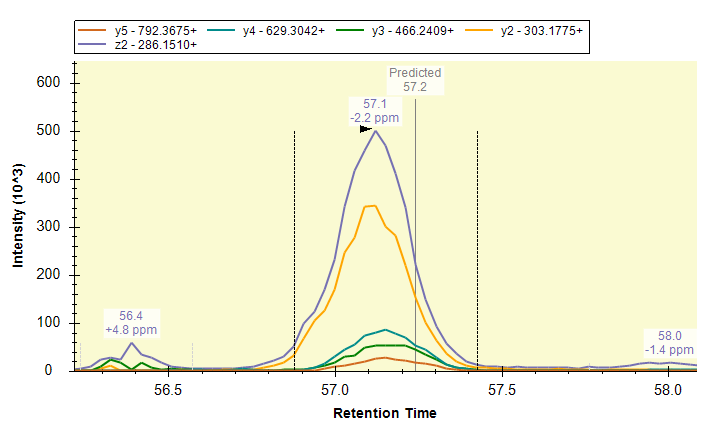


Annotated MS/MS spectrum for doubly charged GFYYYQR, matched for MS feature of triply charged “995.44_995.47_3”.

Annotated MS/MS spectrum for doubly charged SVEVNFTESLLR, matched for MS feature of doubly charged “1392.72_1392.75_3”.

Annotated MS/MS spectrum for doubly charged SIAQYWLGC[+57.021464]PAPGHL, matched for MS feature of doubly charged “1668.8_1668.83_11”.
